# Unravelling pathways downstream Sox6 induction in K562 erythroid cells by proteomic analysis

**DOI:** 10.1038/s41598-017-14336-6

**Published:** 2017-10-26

**Authors:** Gloria Barbarani, Antonella Ronchi, Margherita Ruoppolo, Lucia Santorelli, Robert Steinfelder, Sudharshan Elangovan, Cristina Fugazza, Marianna Caterino

**Affiliations:** 1 Dipartimento di Biotecnologie e Bioscienze, Università degli studi di Milano-Bicocca, Milan, Italy; 2 Dipartimento di Medicina Molecolare e Biotecnologie Mediche, Università degli Studi di Napoli, “Federico II”, Naples, Italy; 3 CEINGE Biotecnologie Avanzate, Naples, Italy; 4 Source BioScience, Nottingham Business Park, Nottingham, United Kingdom; 5 Present Address: Fred Hutchinson Cancer Research Center, Seattle, USA; 6 Present Address: King Abdullah University of Science and Technology, Thuwal, Saudi Arabia

## Abstract

The Sox6 transcription factor is crucial for terminal maturation of definitive red blood cells. Sox6-null mouse fetuses present misshapen and nucleated erythrocytes, due to impaired actin assembly and cytoskeleton stability. These defects are accompanied with a reduced survival of Sox6^−/−^ red blood cells, resulting in a compensated anemia. Sox6-overexpression in K562 cells and in human primary *ex vivo* erythroid cultures enhances erythroid differentiation and leads to hemoglobinization, the hallmark of erythroid maturation. To obtain an overview on processes downstream to Sox6 expression, we performed a differential proteomic analysis on human erythroid K562 cells overexpressing Sox6. Sox6-overexpression induces dysregulation of 64 proteins, involved in cytoskeleton remodeling and in protein synthesis, folding and trafficking, key processes for erythroid maturation. Moreover, 43 out of 64 genes encoding for differentially expressed proteins contain within their proximal regulatory regions sites that are bound by SOX6 according to ENCODE ChIP-seq datasets and are possible direct SOX6 targets. SAR1B, one of the most induced proteins upon Sox6 overexpression, shares a conserved regulatory module, composed by a double SOX6 binding site and a GATA1 consensus, with the adjacent SEC24 A gene. Since both genes encode for COPII components, this element could concur to the coordinated expression of these proteins during erythropoiesis.

## Introduction

Twenty Sox transcription factors exist in mice and humans^1,2^. They feature a Sry-type high-mobility-group (HMG box) DNA binding domain. They act as architectural proteins by assembling on chromatin multiprotein complexes, thus regulating various developmental and differentiation processes. Within this general frame, *Sox6, Sox5* and *Sox13* form the Sox D subfamily, being *Sox6* and *Sox5* very similar. Sox6 is highly expressed in a wide range of tissues throughout life^3–8^. In the adult mouse and human tissues, *Sox6* is expressed in brain, heart, lung, liver, spleen, pancreas, skeletal muscle, kidney and testis. By interacting with *Sox5, Sox6* participates in the activation of chondrocyte-specific genes, in concert with the activator *Sox9* (commonly referred to as the “Sox trio”)^5,9^.

*Sox6* is crucial for terminal erythroid maturation of definitive red blood cells (RBCs)^8,10,11^. In erythropoiesis, committed progenitors progressively differentiate into burst-forming–unit erythroid cells (BFU-E) and colony forming–unit erythroid (CFU-E) cells. CFU-E, in turn, gives rise to pro-erythroblasts, erythroblasts and finally to mature, enucleated red blood cells^8,12–14^. Erythroid differentiation involves the progressive activation of erythroid specific genes, including those encoding for specific RBCs membrane cytoskeleton components and for globin chains, which represent about 95% of RBCs protein content^12,15^. During their terminal maturation, erythroblasts progressively condense their nucleus, which is ultimately extruded together with most organelles. The resulting reticulocyte contains large amount of erythroid-specific mRNAs (most of them encoding for globin chains and for specific membrane glycoproteins), actively translated to sustain protein synthesis until the loss of ribosomes, which coincides with the formation of the typical biconcave red blood cell^16,17^.

*Sox6*-null mouse fetuses and pups present misshapen and nucleated red blood cells, possibly reflecting defects in actin assembly and cytoskeleton stability^8^. As a consequence, Sox6^−/−^ RBCs have a reduced survival, finally resulting in a compensated anemia. Beside its role in erythroid maturation under physiological and stress conditions^18^, Sox6 contributes to the silencing of embryonic globin genes; in mouse, it directly silences the embryonic εy-globin gene in definitive erythroid cells by binding to the εy promoter^13,14^; in humans, it cooperates with BCL11A-XL in silencing ε and γ -globin expression^19^. Importantly, Sox6 overexpression in K562 cells and in human primary *ex vivo* erythroid cultures enhances erythroid differentiation and a general hemoglobinization^11^, the main hallmark of erythroid terminal maturation. Overall, these data point to a critical role of SOX6 for cell survival, proliferation and terminal maturation in both normal and stress erythropoiesis. Despite these evidences, little is known about SOX6 effectors in erythroid cells. With the aim to unravel pathways and processes downstream to SOX6 induction, including both direct and un-direct targets, we performed a differential proteomic analysis on human erythroid K562 cells overexpressing Sox6.

Here we show that Sox6 overexpression is associated with a significant change in proteins controlling cytoskeleton remodeling and protein trafficking, two important processes for erythroid terminal differentiation.

## Results

### Comparative proteomic analysis

**Figure 1:**
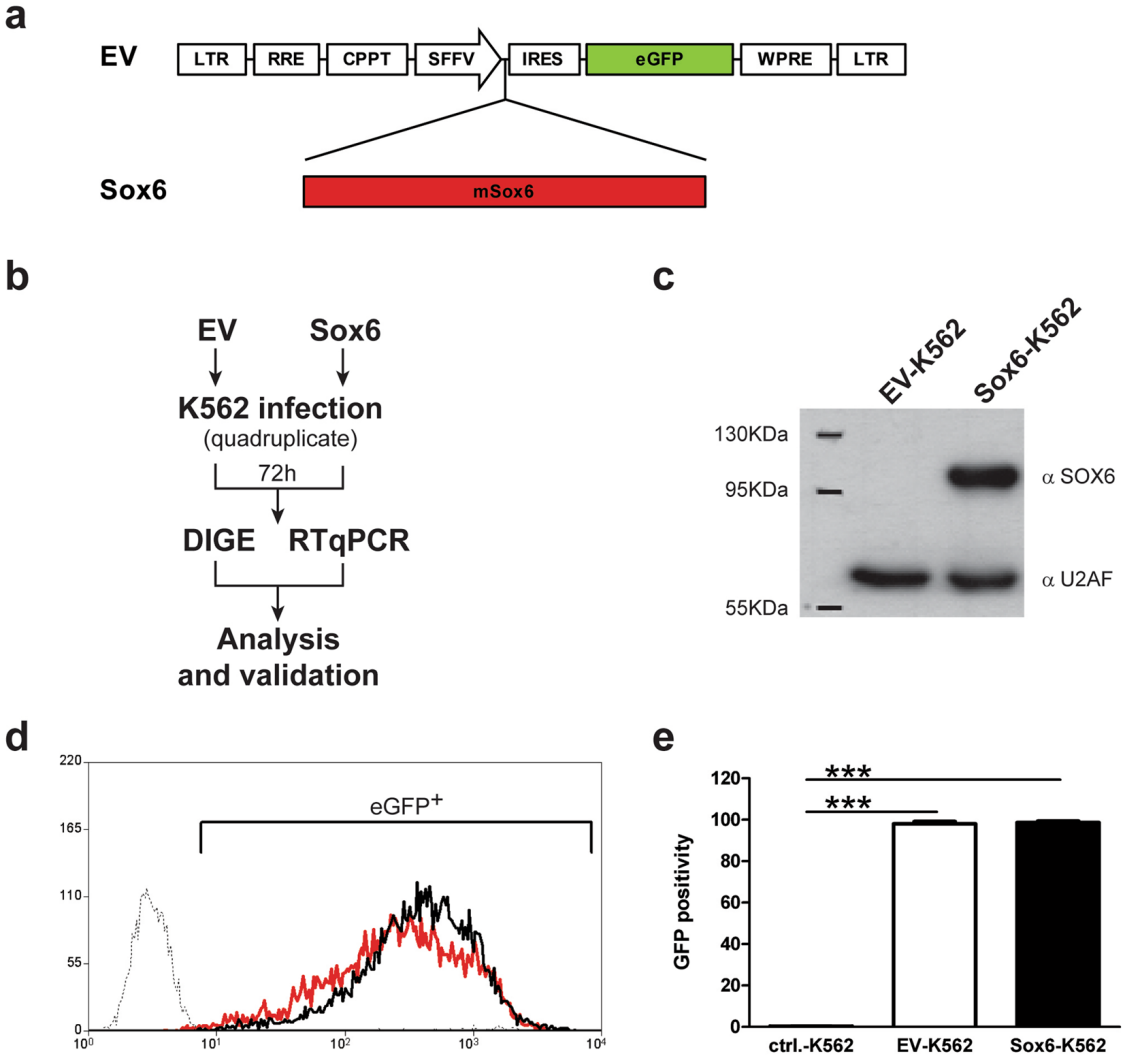
(**a**) Schematic representation of the lentiviral vector used in this study (modified from ref.^11^). (**b**) Outline of the experimental design. (**c**) Representative Western Blot showing the expression of Sox6 protein in Empty vector (EV-K562) and in Sox6-K562 total protein extracts. U2AF (U2 auxiliary factor): protein loading control. (**d**) Representative Flow cytometry (FC) analysis on eGFP to assess the percentage of infected cells. (**e**) Histogram representing the average of the eGFP positivity in transduced vs untransduced control cells (n = 4; error bars: SEM; ***P < 0.001).

**Figure 2:**
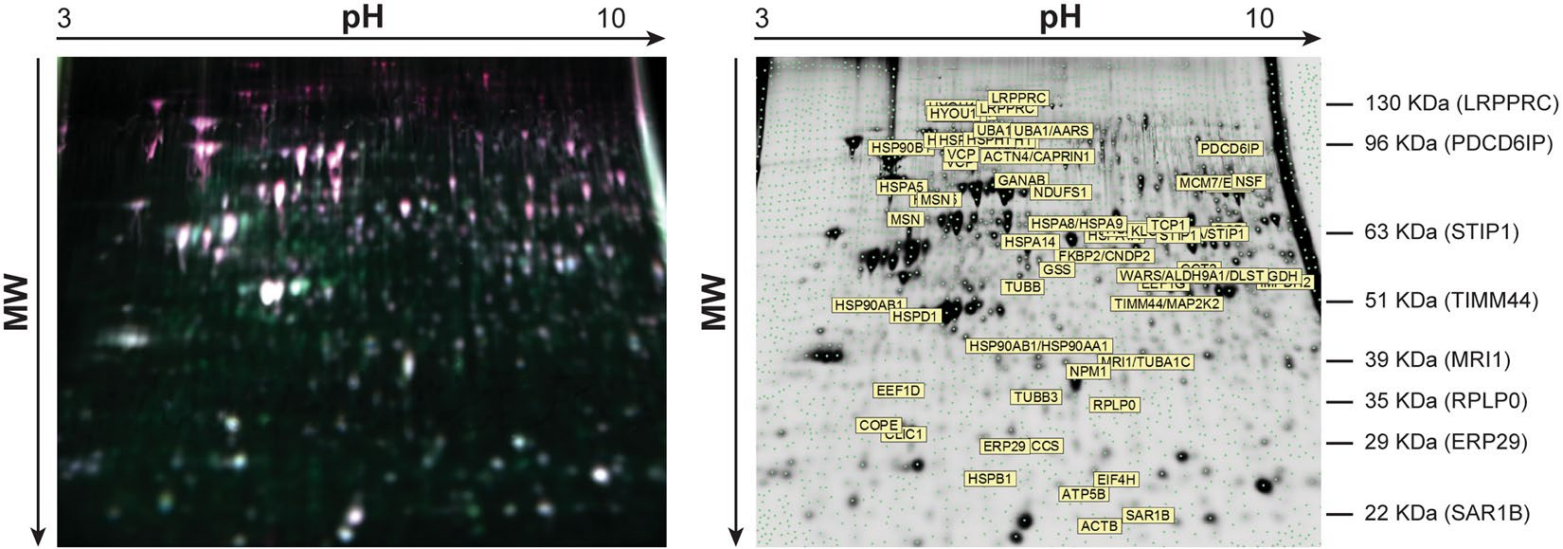
Representative 2D gel (run: 24 cm pH 3–10 NL in the first dimension and 10% SDS PAGE in the second dimension). Total cellular extracts were used. (**a**) Analytical gel image overlay of EV-K562, labelled with Cy5, Sox6-K562, labelled with Cy3 and pool standard labeled with Cy2. (**b**) Preparative gel, stained in Sypro Ruby, showing deregulated proteins. The molecular weight of some reference proteins is indicated. Please note that separation was carried out so that proteins below 20 KDa run out of the gel. Globin chains (around 17 KDa) are therefore not included in the gel.

A comparative proteomic analysis was carried out in order to define protein expression profiles affected by Sox6 overexpression in erythroid K562 cells (Fig. 1). We compared total protein extracts obtained from K562 cells and from the same cells overexpressing Sox6. The experiment was performed in quadruplicate on four pairs of samples from cells transduced with either the Sox6-overexpressing vector (Sox6-K562) or the corresponding Empty Vector (EV-K562) as a control, to ensure statistical replication (Fig. 1b). In all experiments Sox6 was indeed overexpressed (Fig. 1c) and cells were infected at >97% (Fig. 1d–e), thus making any further purification step unnecessary. The set of fluorescent scans is shown in Supplementary Fig. S1. The quantitative analysis was performed so that only proteins up- or down-regulated in all four analysed sample pairs (Sox6-K562 vs EV-K562) were taken into account. The semi-preparative gel was carried out using a major amount of total protein extract in order to allow the subsequent mass spectrometry-based protein identification. The excised and identified spots are shown in Fig. 2b.

We identified 64 differentially expressed proteins. Up-regulated and down-regulated proteins are reported in Table 1. For each protein, the spot number, p-value, fold change, protein description, gene official symbol, Swiss-Prot Accession Number, Gene ID, theoretical Molecular Weight and pI are indicated. Supplementary Table S1 shows the details of mass spectrometric identification. In some cases, the same protein was identified in more than one spot. This could be due to posttranslational modifications that affect proteins migration. The identification of posttranslational modification was out of the scope of this paper.

**Table 1:** Differentially espressed proteins in Sox-6 overexpressing K562 cells.

n SPOT	*p-value*	Folde-change	PROTEIN DESCRIPTION	Official Gene Symbol	Swiss Prot. code	GENE ID	Teorethical MW (KDa)	Teorethical pI
2449	0,0027	2,5	ATP synthase, H+ transporting, mitochondrial F1 complex, beta polypeptide	ATP5B	P06576	506	56,6	5,3
1682	0,0071	2,4	heat shock protein 90kDa alpha (cytosolic), class B member 1	HSP90AB1	Q96KP4	3326	83,2	5,0
1902	0,0087	2,1	heat shock 60kDa protein 1 (chaperonin)	HSPD1	P10809	3329	61,0	5,7
2601	0,012	2,0	SAR1 gene homolog B (S. cerevisiae)	SAR1B	Q9Y6B6	51128	22,4	5,8
2049	0,015	1,8	eukaryotic translation elongation factor 1 delta (guanine nucleotide exchange protein)	EEF1D	P29692	1936	31,1	5,0
2471	0,001	1,8	actin, beta	ACTB	P60709	60	41,7	5,3
2420	0,003	1,7	heat shock 27kDa protein 1	HSPB1	P04792	3315	22,8	6,0
1962	0,003	1,6	nucleophosmin (nucleolar phosphoprotein B23, numatrin)	NPM1	P06748	4869	32,6	4,6
1892	0,004	1,5	heat shock protein 90kDa alpha (cytosolic), class B member 1	HSP90AB1	Q96KP4	3326	83,2	5,0
			heat shock protein 90kDa alpha (cytosolic), class A member 1	HSP90AA1	P07900	3320	85,0	5,0
1872	0,0011	1,4	methylthioribose-1-phosphate isomerase homolog (S. cerevisiae)	MRI1	Q9BV20	84245	39,2	6,0
			tubulin, alpha 1c	TUBA1C	Q9BQE3	84790	50,0	5,0
2140	0,027	1,4	tubulin, beta 3	TUBB3	Q13509	10309	50,4	4,8
1567	0,022	1,4	heat shock protein 90kDa alpha (cytosolic), class B member 1	HSP90AB1	Q96KP4	3326	83,2	5,0
1598	0,0022	1,4	tubulin, beta	TUBB	P07437	203068	49,7	4,8
1131	0,039	1,4	kinesin light chain 4	KLC4	Q9NSK0	89953	68,6	5,8
2391	0,012	1,4	eukaryotic translation initiation factor 4H	EIF4H	Q15056	7458	27,4	6,7
1526	0,034	1,3	glutathione synthetase	GSS	P48637	2937	52,4	5,7
2298	0,00042	1,3	copper chaperone for superoxide dismutase	CCS	O14618	9973	29,0	5,3
2316	0,033	1,3	chloride intracellular channel 1	CLIC1	O00299	1192	27,0	5,1
2318	0,0039	1,2	endoplasmic reticulum protein 29	ERP29	P30040	10961	29,0	6,8
			prohibitin	PHB	P35232	5245	29,8	5,6
2173	0,038	1,2	coatomer protein complex, subunit epsilon	COPE	O14579	11316	34,5	5,0
2014	0,004	1,2	ribosomal protein, large, P0	RPLP0	P05388	6175	34,3	5,7
976	0,04	−1,2	glycyl-tRNA synthetase	GARS	P41250	2617	83,1	6,6
980	0,012	−1,2	NADH dehydrogenase (ubiquinone) Fe-S protein 1, 75kDa (NADH-coenzyme Q reductase)	NDUFS1	P28331	4719	79,5	5,9
1080	0,028	−1,2	heat shock 70kDa protein 9 (mortalin)	HSPA9	P38646	3313	73,7	5,9
			heat shock 70kDa protein 8	HSPA8	P11142	3312	70,9	5,4
1273	0,017	−1,2	t-complex 1	TCP1	P17987	6950	60,3	5,8
1376	0,012	−1,2	desmoplakin	DSP	P15924	1832	331,7	6,4
1438	0,01	−1,2	3-oxoacid CoA transferase 1	OXCT1	P55809	5019	56,2	7,1
			phosphoglycerate dehydrogenase	PHGDH	O43175	26227	56,6	6,3
1456	0,013	−1,2	tryptophanyl-tRNA synthetase	WARS	P23381	7453	53,1	5,8
			aldehyde dehydrogenase 9 family, member A1	ALDH9A1	P49189	223	53,8	5,7
			dihydrolipoamide S-succinyltransferase (E2 component of 2-oxo-glutarate complex)	DLST	P36957	1743	48,7	9,1
1770	0,035	−1,2	translocase of inner mitochondrial membrane 44 homolog (yeast)	TIMM44	O43615	10469	51,3	8,3
			mitogen-activated protein kinase kinase 2	MAP2K2	P36507	5605	44,4	6,1
1382	0,0076	−1,2	heat shock 70kDa protein 14	HSPA14	Q0VDF9	51182	54,8	5,4
1400	0,017	−1,3	FK506 binding protein 4, 59kDa	FKBP4	QO2790	2288	51,8	5,4
			CNDP dipeptidase 2 (metallopeptidase M20 family)	CNDP2	P08238	55748	52,8	5,7
631	0,017	−1,3	heat shock 70kDa protein 4	HSPA4	P34932	3308	94,0	5,1
601	0,0094	−1,3	ubiquitin-like modifier activating enzyme 1	UBA1	P22314	7317	118,0	5,5
			alanyl-tRNA synthetase	AARS	P49588	16	106,8	5,3
745	0,023	−1,3	valosin-containing protein	VCP	P55072	7415	89,3	5,1
1435	0,041	−1,3	chaperonin containing TCP1, subunit 2 (beta)	CCT2	P78371	10576	57,5	6,0
1013	0,027	−1,3	heat shock 70kDa protein 5 (glucose-regulated protein, 78kDa)	HSPA5	P11021	3309	72,3	5,1
955	0,0061	−1,3	minichromosome maintenance complex component 7	MCM7	P33993	4176	81,3	6,1
			ezrin	EZR	P15311	7430	64,0	5,9
1035	0,028	−1,4	moesin	MSN	P26038	4478	68,0	6,1
1052	0,00011	−1,4	moesin	MSN	P26038	4478	67,8	6,1
1162	0,007	−1,4	heat shock 70kDa protein 1 A	HSPA1A	P08107	3303	70,1	5,5
1664	0,0023	−1,4	eukaryotic translation elongation factor 1 gamma	EEF1G	P26641	1937	50,1	6,2
705	0,0078	−1,4	heat shock protein 90 kDa beta (Grp94), member 1	HSP90B1	P14625	7184	92,5	4,8
1278	0,054	−1,4	chaperonin containing TCP1, subunit 3 (gamma)	CCT3	P49368	7203	60,5	6,1
1469	0,028	−1,4	IMP (inosine monophosphate) dehydrogenase 2	IMPDH2	P12268	3615	55,8	6,4
983	0,016	−1,4	N-ethylmaleimide-sensitive factor	NSF	P46459	4905	82,6	6,5
781	0,0061	−1,4	programmed cell death 6 interacting protein	PDCD6IP	Q8WUM4	10015	96,0	6,1
1289	0,061	−1,4	stress-induced-phosphoprotein 1	STIP1	P31948	10963	62,6	6,4
1236	0,029	−1,4	lamin A/C	LMNA	P02545	4000	74,1	6,6
			stress-induced-phosphoprotein 1	STIP1	P31948	10963	62,6	6,4
1387	0,026	−1,4	3-hydroxy-3-methylglutaryl-Coenzyme A synthase 1 (soluble)	HMGCS1	Q01581	3157	57,3	5,2
1464	0,069	−1,4	glucose-6-phosphate dehydrogenase	G6PD	P11413	2539	59,3	6,4
675	0,015	−1,4	actinin, alpha 4	ACTN4	O43707	81	104,9	5,3
			cell cycle associated protein 1	CAPRIN1	Q14444	4076	78,4	5,1
803	0,031	−1,4	valosin-containing protein	VCP	P55072	7415	89,3	5,1
620	0,0057	−1,5	heat shock 70 kDa protein 4	HSPA4	P34932	3308	94,3	5,1
			heat shock 105kDa/110kDa protein 1	HSPH1	Q92598	10808	97,0	5,3
1130	0,082	−1,5	heat shock 70 kDa protein 8	HSPA8	P11142	3312	71,0	5,4
670	0,034	−1,5	alanyl-tRNA synthetase	AARS	P49588	16	106,8	5,3
594	0,003	−1,5	ubiquitin-like modifier activating enzyme 1	UBA1	P22314	7317	117,8	5,5
			alanyl-tRNA synthetase	AARS	P49588	16	106,8	5,3
608	0,0088	−1,5	alanyl-tRNA synthetase	AARS	P49588	16	106,8	5,3
626	0,007	−1,5	heat shock 70 kDa protein 4	HSPA4	P34932	3308	94,3	5,1
1053	0,034	−1,6	heat shock 70 kDa protein 5 (glucose-regulated protein, 78 kDa)	HSPA5	P11021	3309	72,3	5,1
697	0,035	−1,6	glucosidase, alpha; neutral AB	GANAB	Q14697	23193	107,0	5,7
624	0,015	−1,6	heat shock 105 kDa/110 kDa protein 1	HSPH1	Q92598	10808	97,0	5,3
388	0,024	−1,7	leucine-rich PPR-motif containing	LRPPRC	P42704	10128	158,0	5,8
413	0,043	−1,8	hypoxia up-regulated 1	HYOU1	Q94L1	10525	111,3	5,2
603	0,0089	−1,8	alanyl-tRNA synthetase	AARS	P49588	16	106,8	5,3
401	0,024	−1,9	hypoxia up-regulated 1	HYOU1	Q9Y4L1	10525	111,3	5,2
410	0,025	−1,9	leucine-rich PPR-motif containing	LRPPRC	P42704	10128	158,0	5,8
414	0,033	−2,0	hypoxia up-regulated 1	HYOU1	Q9Y4L1	10525	111,3	5,2

The whole set of differentially expressed proteins was analysed using the STRING functional protein interaction networks (http://string-db.org/).

**Figure 3:**
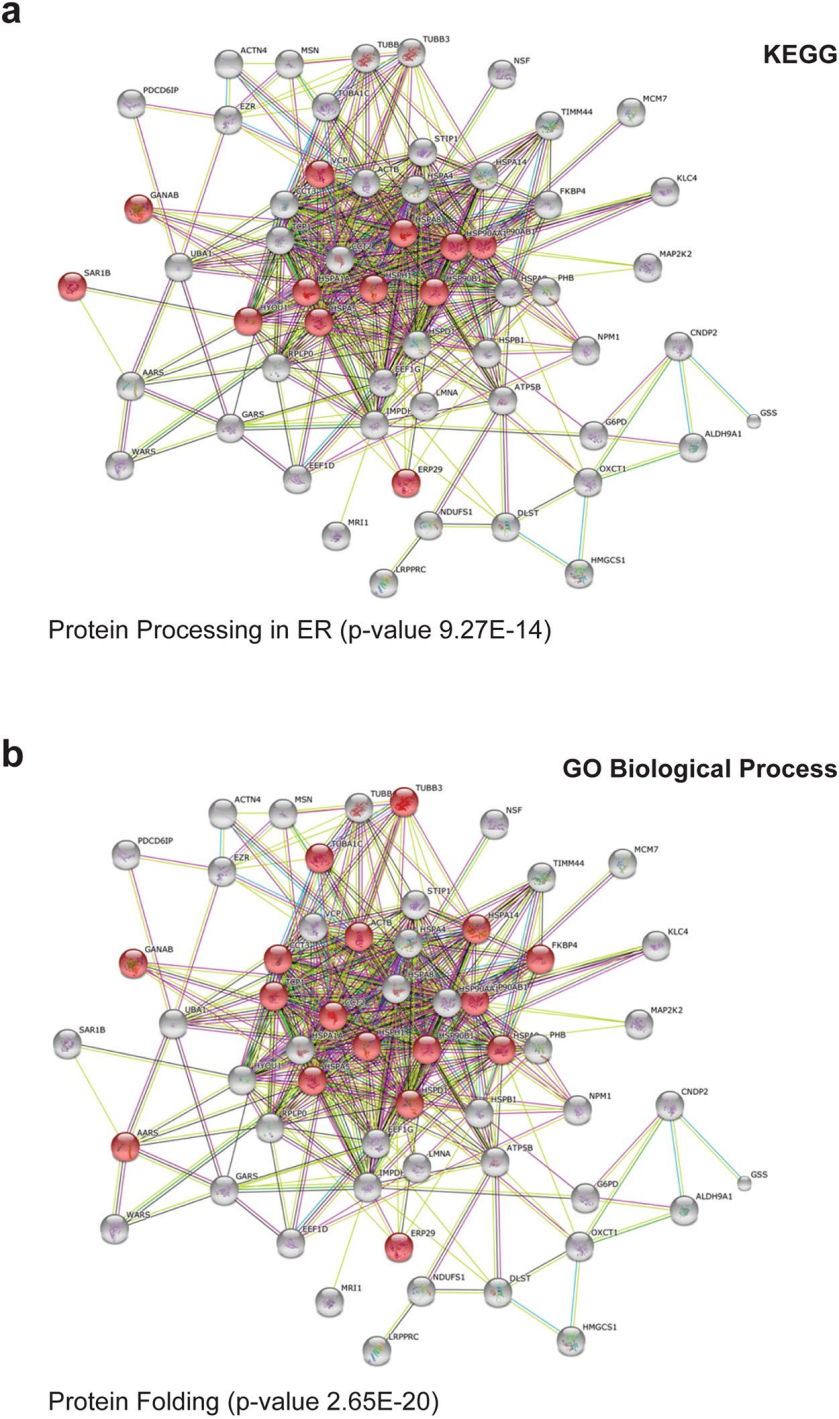
The network distributions of the 64 differentially expressed proteins were explored using STRING software. (**a**) According to “Kyoto Encyclopedia of Genes and Genomes” (KEGG) database, the most significant pathway is “protein processing in endoplasmic reticulum (ER)” (number of genes: 12, p-value 9.27E^−14^). (**b**) According to “Gene Ontology” (GO), the most significant biological process is “protein folding” (number of genes: 17, p-value 2.65E^−20^). In panels a and b all proteins involved in the networks are shown. Amongst them, proteins identified as differentially expressed in our study are in red.

Table 2 shows the statistical significant pathways (*p* < 0.001) predicted by Kyoto Encyclopedia of Genes and Genomes (KEGG), with the most significant pathway being “Protein processing in endoplasmic reticulum” (number of genes 12, p-value 9.27E^−14^). Figure 3a shows the graphical representation of this pathway with the proteins identified in this study highlighted in red. This pathway drew our attention because a mutation in a component (SEC23a) of the COPII complex, responsible for the anterograde transport from ER to Golgi has been recently involved in the pathogenesis of congenital diserythropoietic anemia, type II. Of interest, in our list of dysregulated proteins (Table 1) we found SAR1B (nSPOT 2601, fold change 2.0), a COPII component, and COPE (nSPOT 2173, fold change 1.2), a member of the COPI anterograde complex, thus suggesting an increased protein trafficking in both directions in K562 cells overexpressing Sox6.

**Table 2:** KEGG clustering analysis.

Pathway	Number of Genes	p-value
Protein processing in endoplasmic reticulum	12	9.27E-14
Metabolic pathways	12	2.23E-4
Aminoacyl-tRNA biosynthesis	3	3.3E-4
Synthesis and degradation of ketone bodies	2	3.42E-4
Amminoacid metabolism	6	3.53E-4
Leukocyte transendothelial migration	4	4.34E-4
Regulation of actin cytoskeleton	5	4.85E-4
Butanoate metabolism	2	2.98E-3
Histidine metabolism	2	3.22E-3
MAPK signaling pathway	4	7.91E-3
Glutathione metabolism	2	1.03E-2

Table 3 shows the predicted most significant pathways according to Gene Ontology (GO) Biological Process. Amongst them, there is protein folding (number of genes 16, p-value 2.65E^−20^), an important process in late phases of erythropoiesis. Figure 3b shows the graphical representation of the pathway, with in red the protein identified by Gene Ontology. A high number of chaperone proteins, which play an important role in different areas of erythropoiesis, was dysregulated upon Sox6 overexpression. Amongst them, eight proteins (HSPA1A, HSPA4, HSPA5, HSPA8, HSPA9, HSPA14, HYOU1, HSPH1/HSP105-100), belonging to the HSP70 family, are downregulated whereas other HSPs (HSP90AB1, HSP90AA1, HSPD1/HSP60, HSPB1/HSP27) are increased (Table 1).

**Table 3:** GO Biological process clustering analysis.

Term	Number of Genes	p-value
Protein folding	16	2.65E-20
Metabolic process	31	2.26E-10
Cellular protein complex assembly	19	9.47E-9
Vesicle-mediated transport	15	6.11E-7
Endoplasmic reticulum unfolded protein response	6	9.86E-7
Intracellular transport	15	3.13E-6
Cellular component biogenesis	18	5.79E-6
Regulation of apoptotic process	15	5.96E-6
Macromolecule localization	18	9.24E-6
Peptide biosynthetic process	8	1.15E-5
Endocytosis	8	1.03E-4
Mitochondrion organization	7	1.92E-4
tRNA aminoacylation for protein translation	3	3.07E-4
Movement of cell	12	3.28E-4
Glutathione metabolic process	3	3.3E-4

### Direct vs Indirect Sox6 targets

**Figure 4:**
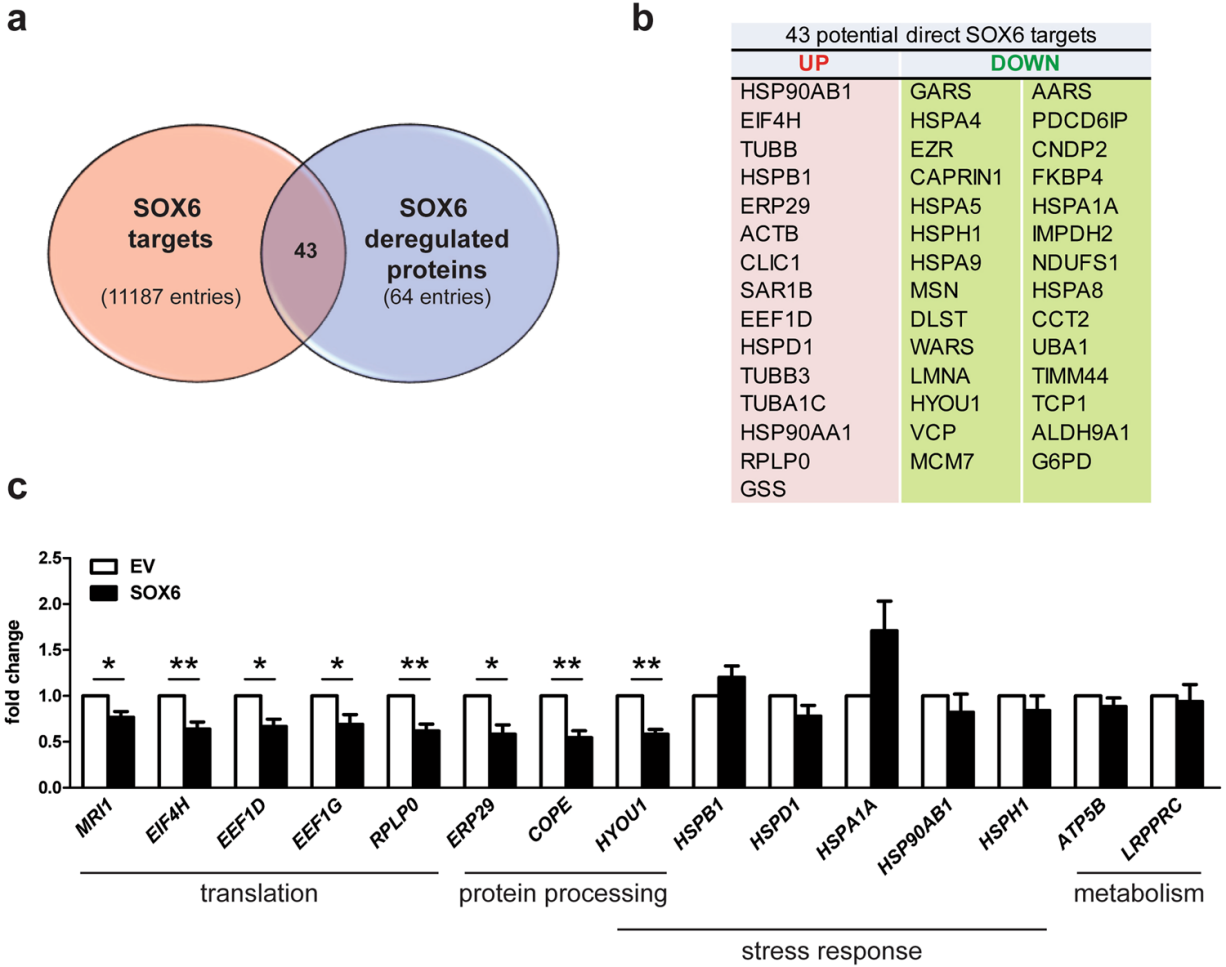
(**a**) Venn diagram merging the list of candidate direct SOX6 targets with that of the differentially expressed proteins identified by the proteomic analysis. Potential direct Sox6 target genes were here defined as genes bound by SOX6 in their −/+5 kb region with respect to their transcriptional starts sites in Sox6-ChIP-seq data from ENCODE (see Materials and methods for details). (**b**) 43 out of 64 genes coding for proteins increased (up) or decreased (down) upon SOX6 overexpression contain sequences bound by Sox6 in their proximal regulatory regions. (**c**) RT-qPCR on some of the above genes, expressed in fold change, with the expression level in EV-K562 set equal to 1 (n = 4; error bars: SEM; *P < 0.05, **P < 0.01). The main process in which these genes are involved is indicated below the panel. HYOU1 is a member of the HSP70 mainly involved in the unfolding protein response.

**Figure 5:**
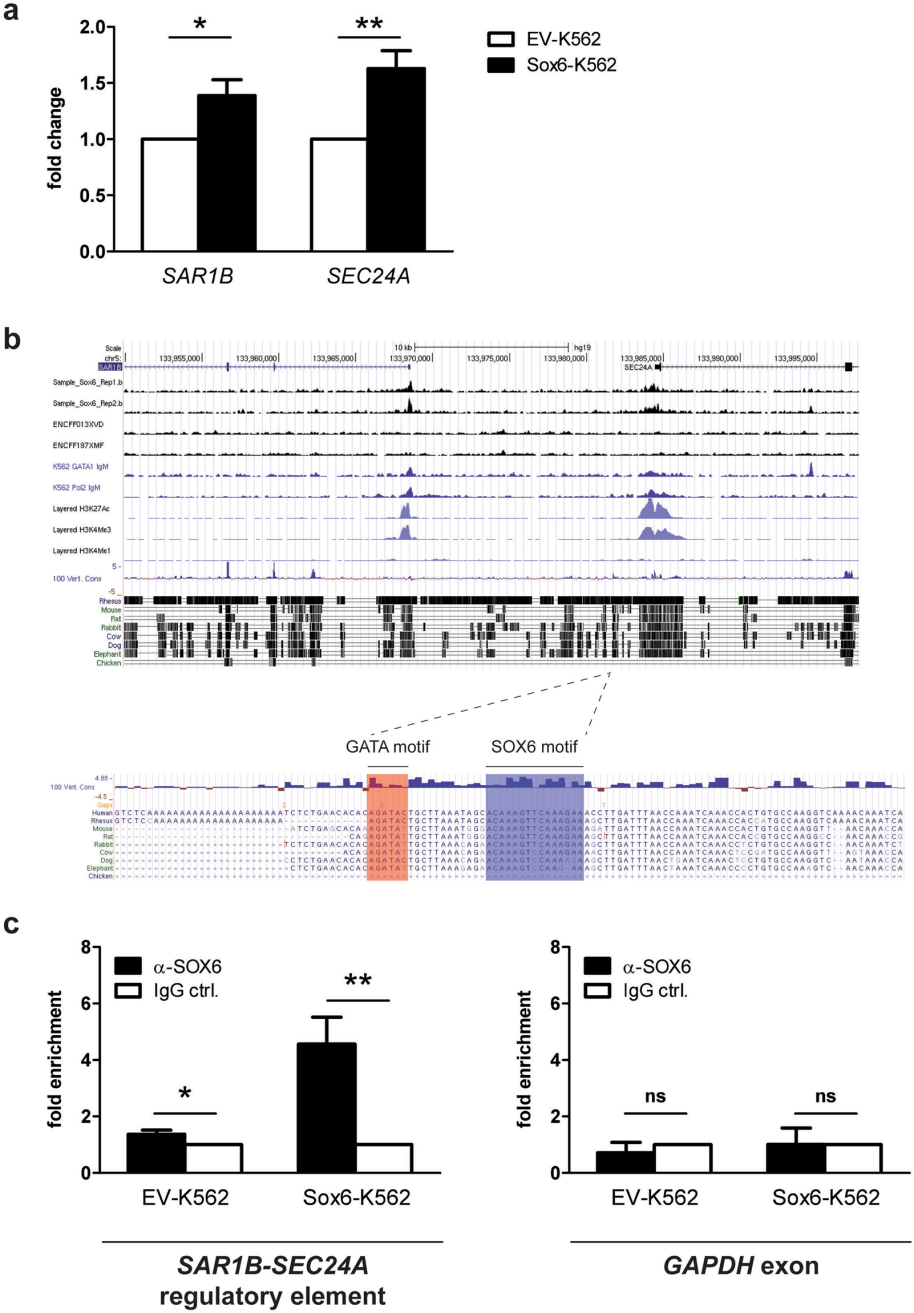
(**a**) RT-qPCR showing the expression level of *SAR1B* and *SEC24A*. EV: cells transduced with the empty vector; Sox6: cells transduced with the Sox6 overexpressing vector (n = 7; error bars: SEM; *P < 0.05, **P < 0.01). (**b**) Screenshot of the human chromosome 5 region containing *SAR1B* and *SEC24A* genes from UCSC genome browser (https://genome.ucsc.edu). Two replicates of SOX6 ChIP-seq data (on K562 and HepG2), and GATA1, RNA polymerase II, H3K27Ac, H3K4me3 and H3KMe1 ChIP-seq data on K562 cells are shown. Evolutionary conservation of the regulatory module composed by a double SOX6 (blue) and a single GATA1 (red) binding sites lying between *SAR1B* and *SEC. 24A* genes (chr5:133981706-133981762 on the Hg19 UCSC annotated genome). (**c**) Chromatin Immunoprecipitation in K562 cells overexpressing Sox6 demonstrates the *in vivo* SOX6 binding to the SOX6 double site region identified in panel b. Data are shown as a fold enrichment over the IgG control (n = 3; error bars: SEM, *P < 0.05, **P < 0.01). An anti-SOX6 antibody was used to immunoprecipitate SOX6. Isotypic IgG were used as a control. In the right panel an unrelated sequence (GAPDH exon) was tested as a further negative control.

The main goal of our work was to have a global overview on processes downstream to Sox6 expression in erythroid cells, without discriminating direct versus indirect Sox6 targets. However, to have a first glance on this point, we selected genes having SOX6 binding peaks in their −/+5 kb region with respect to their transcriptional starting sites (Encode SOX6-ChIP-seq data, ENCSR788RSW) and we intersected this list with the group of dysregulated proteins in our proteomic experiment. As shown in Fig. 4a, the resulting Venn diagram suggests that 43/64 genes encoding for differentially expressed proteins contain sequences bound by SOX6 in K562 cells in their proximal regulatory regions and are thus possible direct SOX6 targets. The 43 potential direct SOX6 targets are listed in Fig. 4b. To reinforce the notion that these genes could be relevant for the erythroid lineage, we looked for GATA1 peaks in the same regulatory regions, according to ENCODE ChIP datasets (Fig. 5 and Supplementary Fig. S2). In fact, GATA1 is the master regulator of erythroid commitment and differentiation. All the 43 genes present GATA1 bound sites within −/+1 kb from the Sox6 occupied sites, confirming our hypothesis.

### Transcriptional vs post-transcriptional changes

As a next step, we analyzed the expression of some of the above genes chosen as representative of the different categories (i.e. translation, protein processing, stress response, metabolism, Fig. 4c) in the same cells used for the proteomic analysis (Fig. 1b). RTqPCR shows that the expression of genes coding for proteins involved in translation/protein processing at different level (MRI/EIF2B-like, EIF4H, EEF1D, EEF1G, RPLP0, ERP29, COPE, HYOU1) are significantly reduced (from 23% to 42% compared to the control). SAR1B and SEC24A are increased (see below). Finally, some of the tested RNAs do not change significantly. In particular, mRNAs corresponding to the most dysregulated “metabolic” proteins (ATP5B: increased; LRPPRC: decreased), do not change their expression level and do not present any potential Sox6 binding site in their proximal regulatory elements, suggesting that they are indirect downstream Sox6 effectors. Overall, these data are in line with the notion that Sox6 forces cells towards late erythropoiesis stages, when transcription globally declines along with progressive nuclear condensation.

### The expression of *SAR1B* and *SEC24A* increases in K562 cells overexpressing Sox6

Notably, SAR1B is one of the most induced proteins upon Sox6 overexpression in K562 (nSPOT 2601, Fold change + 2.0, Table 1) and is also upregulated at RNA level (Fig. 5a). We noticed that *SAR1B* lies on chromosome 5, about 15 kb apart from *SEC24A* and that the two genes are transcribed in opposite orientation. Because SAR1B and SEC24A are both part of the COPII complex, involved in protein folding and trafficking, this evidence raises the possibility that the two genes could be co-regulated to ensure their coordinated expression during erythropoiesis. Indeed, RTqPCR analysis revealed that both genes are significantly upregulated upon Sox6 overexpression (Fig. 5a), although SEC24A could not be detected in the list of dysregulated proteins shown in Table 1, possibly because its natural level of expression falls below the detection limits of the 2D-DIGE technique (~0.25 ng protein).

### In K562, Sox6 binds *in vivo* to a double SOX6 consensus lying between *SAR1B* and *SEC24A*

The above results suggest that *SAR1B* and *SEC24A* could be direct targets of Sox6. To strengthen this hypothesis, we searched the region between the two genes for a possible conserved SOX6 binding site. As shown in Fig. 5b, we found a conserved double SOX6 binding site at chr5:133981706-133981762 on the Hg19 UCSC annotated genome. Chromatin Immunoprecipitation indeed confirmed the ability of SOX6 to bind this target in Sox6-overexpressing K562 cells (Fig. 5c). Moreover, we noticed that, adjacent to the double Sox6 site lies a conserved “AGATA” site, a perfect consensus for GATA1, the major erythroid transcription factor. This site can be bound *in vivo* by GATA1 according to ENCODE data (Fig. 5b). The conservation of these adjacent elements indicates that this region could act as an erythroid-specific regulatory element shared by *SAR1B* and *SEC24A*, as suggested by its absence in the non-erythroid hepatocellular HepG2 cell line (Fig. 5b).

## Discussion

We report the identification of dysregulated proteins in the human erythroid cell line K562 upon lentiviral-mediated Sox6 overexpression. We identified a set of 64 proteins related to several cell processes (Table 1). The biological processes predominantly influenced by Sox6 induction are protein metabolism (synthesis, folding and trafficking) and regulation of actin cytoskeleton (Tables 2 and 3).

Proteomic comparative analysis revealed that Sox6 overexpression in K562 cells causes a marked de-regulation of a high number of proteins involved in the “chaperone machinery” as HSP90AB1 (fold change + 2.4) HSPD1 (fold change + 2.0), HSPB1 (fold change + 1.7), HSP90AA1 (fold change + 1.5), ERP29 (fold change + 1.2), TCP1 (fold change −1.2), HSPA9 (fold change −1.2), HSPA14 (fold change −1.2), FKBP4 (fold change −1.3), CCT2 (fold change −1.3), CCT3 (fold change −1.4), HSP90B1 (fold change −1.4), HSPA4 (fold change −1.5), HSPA8 (fold change −1.5), HSPA5 (fold change −1.6), GANAB (fold change −1.6), HSPH1 (fold change −1.6), HYOU1 (fold change −1.8). Molecular chaperones play a key role in different areas of erythropoiesis^20^. Hsp70/HSPA4 and the related protein HSPA9B (alias HSPA9) help to maintain cell survival in the face of apoptotic stimuli that are required for the completion of normal erythropoiesis. Regulated apoptosis is indeed an important mechanism for modulating red cell production according to physiological needs. Erythropoietin, the most important survival factor for erythropoietic development, positively influences HSP70 and HSP9B activities to enhance cell survival^20^. In particular, HSP70 protects GATA1 from caspase-3 cleavage during erythroblasts expansion. However, GATA1 must subsequently decrease to allow terminal maturation^21^.

Of interest, in our study, HSPB1, involved in the degradation of GATA1 in late erythropoiesis, is increased, whereas HSP70 is decreased. Together, these data suggest that the events downstream to Sox6 could contribute to fine tune erythroid differentiation through the regulation of GATA-1 content and activity, by modulating the level of chaperone proteins^22,23^.

Moreover, molecular chaperones regulate hemoglobin homeostasis, a critical aspect of erythrocyte production and function. HSP70 and HSP90/HSP90AA1 are in fact required to generate active heme regulated inhibitor of translation (HRI), which in turn, balances globin synthesis with heme availability^24^. Indeed, aggregates of unbound globin chains (as for α chains in β-thalassemia) or accumulation of abnormal chains (as for HbS in Sickle Cell Disease) pose a serious threat for the integrity of the red blood cells, resulting in hemolysis and in ineffective erythropoiesis, as demonstrated by the above mentioned β-globins disorders^25–27^. Of note, in our analysis, different chaperone proteins show variation in opposite direction, suggesting their specific role in erythroid cells. In these cells, chaperone proteins are likely involved in optimizing protein synthesis of the maturing erythroblast to facilitate the accumulation of hemoglobin and of specific RBCs membrane proteins as well as the degradation of unnecessary proteins. In this view, specific changes in the different chaperones could help to progressively streamline the RBCs protein synthesis repertoire towards the accumulation of large amounts of few specialized proteins (globin chains represent about 95% of the RBCs protein content). Moreover, the decrease in some chaperones (including HSPA5/BiP, and HYOU1) involved in the unfolding protein response (UPR) suggests that the need of synthesizing large amount of proteins could require a relaxation of a strict “quality control” and of the stress response induced by protein synthesis overload. Of note, a high number of proteins changing their level upon Sox6 overexpression, have proteins processing activity within the endoplasmic reticulum (ER), including some of the chaperons mentioned above. Considering that the efficient biosynthesis of RBCs membrane glycoproteins requires a robust ER assembly machinery involving protein translocation, *N*-glycosylation, and protein folding chaperones, this process is a fundamental step of erythropoiesis.

Among ER proteins increased upon Sox6 overexpression, SAR1B shows a significant upregulation (fold change + 2.0, Table 1). Two SAR1-related genes, *SAR1A* and *SAR1B*, are present in mammals, including humans^28,–,30^. In particular, *SAR1B* is activated by Hydroxyurea and its induction increases γ-globin expression in primary CD34^+^ cells^31^. This effect is in contrast with the known role of SOX6 as γ-globin repressor, in cooperation with BCL11A-XL^19^. Since Sox6 enhances the expression of all globins normally expressed by K562 cells, including γ^11^, we speculate that this contrasting effect could be part of a fine-tuning regulatory mechanisms of γ levels in the context of general hemoglobinization stimulated by Sox6.

SAR1 is involved in membrane trafficking machinery^28,32^. SAR1B is in fact a component of coat protein complex II (COPII)–coated vesicles^28,32,33^ that bud from the surface of the ER and transport cargo proteins to the Golgi apparatus. SAR1 proteins initiate vesicle formation on ER membranes through the exchange of GDP for GTP, which induces tight membrane association of SAR1 and the subsequent recruitment of the heterodimeric complex comprising of SEC23/24 COPII components. There are four paralogs for *SEC24* (*SEC24A*, *SEC24B*, *SEC24C* and *SEC24D*) and two paralogs for *SEC23* (*SEC23A* and *SEC23B*) in mammals. SEC23 is a GTPase activating protein (GAP) that stimulates the enzymatic activity of SAR1, whereas SEC24 is the adaptor protein that captures specific cargo into the nascent vesicle. The SAR1-GTP/SEC23/SEC24 “pre-budding” complex in turn recruits the SEC13/SEC31 heterotetramer, which forms the outer layer of the COPII coat, a flexible coat cage that can accommodate various sizes of vesicles, and likely cross-links adjacent pre-budding complexes to complete the vesicle biogenesis process. Mutations in the components of the COPII core trafficking machinery cause human genetic disorders: the Chylomicron Retention Disease (CMRD, mut. in *SAR1B*), the Cranio-lenticulo-sutural dysplasia (CLSD, mut. in *SEC23A*), Congenital Dyserythropoietic Anemia type II (CDA II, mut. in *SEC23B*) and the Combined Deficiency of Factor V and VIII (F5F8D, mut. in *LMAN1* and in MCFD2 Cargo receptors)^28,32,34^. In particular, CDAII mutations in *SEC23B* enlighten the role of COPII in erythroid cells, where impaired processing of glycoproteins results in anisopoikylocytosis, a phenotype partially reminiscent of that of Sox6^−/−^ RBCs.

Although protein trafficking is an ubiquitous cellular mechanism, the specific phenotype of defects in COPII components suggests that the most affected cell types are the ones where large amounts of specific proteins need to be secreted and that the different paralogs genes have different ability to compensate for each other loss in different cell types. This is the case of chondrocytes in CLSD. Since Sox6 is an important transcription factor also for chondrocytes differentiation, this observation suggests that COPII assembly proteins could be important downstream Sox6 effectors in various cell types.

*SAR1B* appears to be a direct SOX6 target, possibly together with *SEC24ASEC24A* lies in proximity of *SAR1B* on chromosome 5, where the two genes are transcribed in opposite orientation. Their expression levels in Sox6-overexpressing K562 cells are concomitantly and significantly increased (Fig. 5). The analysis of the conserved sequences in the region within the two genes revealed the presence of a double Sox potential target site, indeed bound *in vivo* by SOX6 in chromatin immunoprecipitation experiments (Fig. 5c). Adjacent to this element there is an evolutionary conserved *bona fide* GATA1 binding site. These results suggest that *SAR1B* and *SEC24A* could share a common regulatory sequence contributing to their coordinated expression. The dynamics of the binding of these two transcription factors to this region will be the subject of future investigation.

Another class of proteins dysregulated in K562 cells overexpressing Sox6 is that of proteins related to actin cytoskeleton. In our experiment, β-actin shows a fold change of +1.8 (Table 1). Although Sox6^−/−^ mice have almost normal level of β-actin and the defect is mainly in F-actin assembly^8,18^, Sox6 overexpression in K562 shows a clear accumulation of β-actin, suggesting that high levels of Sox6 also promote actin accumulation.

Overall, the pathways downstream to SOX6 in K562 cells identified in this study comprise protein synthesis (at different levels: translation, protein folding and trafficking, amminoacids metabolism, Aminoacyl-tRNA biosynthesis**)** and regulation of actin cytoskeleton. This result suggests that Sox6 overexpression influences two processes crucial for late erythroid maturation: the skewing of the protein synthesis machinery toward a massive production of highly specific proteins and the cytoskeletal reorganization required for preparing erythroblasts for their final maturation, culminating in the nuclear extrusion and in the assembly and stabilization of the highly specialized RBCs membrane.

## Material and Methods

### Cell Cultures

Human erythroleukemic K562 cells were cultured in RPMI 1640 (Lonza) supplemented with 10% heat inactivated fetal bovine serum (Lonza), L-glutamine and antibiotics (Penicillin-Streptomycin 100 U/100 ug/ml) in a humidified 5% CO_2_ atmosphere at 37 °C.

### Sox6 overexpressing vectors

The Sox6 cDNA was cloned upstream to an IRES- eGFP (EmeraldGFP) cassette into a BamH1 blunted site of the CSI lentiviral vector (a kind gift from Prof. T. Enver), under the control of the spleen focus-forming virus (SFFV) promoter, as described elsewhere^10,11^. The resulting vector was checked for the expression of exogenous mRNA and protein in K562 cells (Fig. 1). The corresponding empty vector (EV) was used as a control in all the experiments.

### Western Blot

Western blot was performed in standard conditions. Anti-Sox6 antibody: Abcam AB30455. Anti–U2AF antibody: Sigma U4758.

### Lentiviral Production and transduction experiments

Exponentially growing HEK-293T cells were transfected with jetPEI-TM reagent (Polyplus-Transfection) with the above vectors plus the two packaging plasmids psPAX2 and pMD-VSVG to produce the lentiviral pseudo-particles (www.lentiweb.com). For each virus (Sox6, or EV), 72 h after transfection, the supernatant containing the recombinant particles was collected, filtered and centrifuged at 20,000 g for 8 hours at 4 °C. The viral pellet was re-suspended in PBS and stored in aliquots at −80 °C. Lentiviruses were titrated on HEK-293T cells by measuring the percentage of eGFP^**+**^ cells by flow cytometry (see below). K562 transduction was performed overnight at a multiplicity of infection (MOI) equal to 30. Cell were collected and analyzed 72 h after transduction.

### Flow Cytometry

In all the experiments, the percentage of infection was >97%, as assayed by flow cytometry on eGFP on a Becton-Dickinson FACSCalibur. The Summit V4.3 software was used for the analysis of data.

### Proteomic analysis

DIGE experiments were performed on four biological replicates of cellular extracts from the Sox6-overexpressing cell line (Sox6-K56) and the corresponding four cellular extracts from the control cell line (EV-K562), as previously described^35,36^. Cell lines were homogenized in 0.5 ml of lysis buffer (7 M urea, 2 M thiourea, 4% chaps, 30 mM Tris-HCl pH 7.5) using a Dounce homogenizer. The lysate was precipitated and the protein pellet was solubilized in 100 μL of lysis buffer, composed by 7 M urea, 2 M thiourea, 30 mM Tris-HCl pH 8.5, 4% CHAPS (w/v). According to Ettan DIGE User Manual (18-1173-17 GE Healthcare, Piscataway, NJ) 50 ug of protein extract, to each replicates, were labeled with minimal fluorescent dye Cy2, Cy3 and Cy5^35,36^. The label reaction was stopped by 10 mM L-lysine for 10 min. The labelled protein mixture were fractionated on 18 cm IPG strips with 3–10 NL^36,37^. The IPG strips were focused for a total of 60 kV/h at 20 °C during 18 h. The focused proteins were equilibrated in 6 M urea, 100 mM Tris pH 8.0, 30% glycerol (v/v), 2% SDS, reducted in 0.5% dithiothreitol and carbamidomethylated in 4.5% iodoacetamide. The strips were runned on 10% SDS-PAGE using Ettan Dalt Twelve system (GE Healthcare, Piscataway, NJ) for 18 h at 2 W. The fluorescent images were acquired on Typhoon 9400 Variable Mode Imager (GE Healthcare, Piscataway, NJ) at excitation/emission values of 488/ 520 (Cy2), 532/580 (Cy3), 633/670 nm (Cy5), using 100 µm resolution.

Image files were processed by DeCyder v5.2 software (GE Healthcare) in Batch Processing mode^37,38^. The Differential In-Gel (DIA) module was used to detect and quantify protein spots in a single gel, to define spot boundaries and to normalize the spot volume versus the volume of the relative spot from internal standard; the Biological Variation Analysis (BVA) module was used to match protein-spot between replicate gels^37,38^, to calculate the spot intensity as a mean value of the replicated analysis, and finally to compare the analyzed conditions: cell lines expressing Sox6 and control cells. Student’s t test analysis was used to evaluate statistical differences in spot intensity. Statistically significant protein spot variations (*t*-test: *p* < 0.05) were selected as true positive. The spot matching accuracy was verified by manual inspection. 500 ug of protein extract from all cellular extracts (K562_Sox6 and K562_EV biological replicates) were fractionated on the independent semipreparative 2D-SDS PAGE. The semipreparative gel was fixed in 40% methanol, 10% acetic acid stained in Sypro Ruby dye (Molecular Probes Inc., Eugene, OR), and acquired at an excitation/emission wavelength of 532/610 nm. The selected spots were picked by Ettan Spot Picker (GE Healthcare, Piscataway, NJ) and hydrolyzed using trypsin enzyme at 37 °C overnight. The mass spectrometry analysis was carried out^35,36^ using a LC/MSD Trap XCT Ultra (Agilent Technologies, Palo Alto, CA). The Agilent Data Analysis software provided peak lists used to perform the protein identification by in house Mascot software (http://www.matrixscience.com). The search was obtained using the following parameters: NCBInr database (www.ncbi.nlm.nih.gov); Homo Sapiens as taxonomic origin, trypsin as specific proteolytic enzyme; up to 1 missed cleavage; S-carbamidomethylation as Cys fixed chemical modification; oxidation as variable Met modification; pyro-Glu cyclization as variable Gln N-terminal modification; 200 ppm mass tolerance both on precursor peptide and on fragment mass. The protein identification is accepted only in the presence of at least two peptides, whose mascot score is greater than 38. Mascot ion-score was defined as −10 × Log(P), where P is the probability that the observed event is not random. (p < 0.05)^38^. Protein identification by single peptide mass spectra were not accepted.

### Clustering analysis

The whole group of differentially expressed proteins was analyzed using the ‘STRING: functional protein association networks’ software 7.0 (http://string-db.org/) to evaluate the significant networks and canonical pathways associated with the differentially expressed proteins. STRING is a database of known and predicted protein interactions: the interactions include direct (physical) and indirect (functional) associations. The score of each network or pathway is equal to the negative logarithm of the p-value and represents the likelihood that the assembly of the set of proteins identified in this study is part of significant canonical pathways or networks.

### Identification of candidate SOX6 direct targets

The following data sets available in ENCODE on K562 cells were used: SOX6, ENCSR788RSW; GATA1, ENCSR000EFT; H3K27Ac, ENCSR000AKP; H3K4Me3 (ENCSR000AKU); H3K4Me1, (ENCSR000AKS); polII (ENCSR000EHL). The data set on HepG2 used is ENCSR543BVU. Peaks from MACS2 in the Encode biosamples were annotated against the transcription site annotation of the nearest gene (version NCBI36) by using ChIPpeakAnno (http://bioconductor.org). For analysis, we only considered high scoring (>300) binding sites and regions present in both ENCODE replicates. To select for promoters, binding regions were overlapped with genomic regulatory regions close to promoters, defined as +/−5 kb from transcriptional start sites (TSS) of all annotated transcripts.

### RNA isolation and RTqPCR

Total RNA was extracted with TRI Reagent (Applied Biosystems), treated with RQ1 DNase (Promega) for 30 min at 37 °C and retro-transcribed (High Capacity cDNA Reverse Transcription Kit, Applied Biosystems). Negative control reactions (RT^**−**^) gave no signal. Real time analysis was performed using ABI Prism 7500 (PE Applied Biosystems). Primers were designed to amplify 100 to 150 bp amplicons, on the basis of sequences derived from the Ensembl database (http://www.ensembl.org/). Specific PCR products accumulation was monitored by SYBR Green dye fluorescence in 12 μl reaction volume. Dissociation curves confirmed the homogeneity of PCR products. Primers sequences are listed in Supplementary Table S2.

### Chromatin Immunoprecipitation (ChIP) assay

1 × 10^6^ K562 cells for each Immunoprecipitation reaction were fixed with 1% formaldehyde for 10 minutes at RT and chromatin was sonicated to a size of about 500 bp. Immunoprecipitation was performed after overnight incubation with anti-IgG (SantaCruz) or the anti-Sox6 antibody (Millipore) and subsequent incubation with protein A agarose (Millipore). Immunoprecipitated DNA was then analyzed by amplifying an equivalent of DNA from 10^4^ K562 cells. An unrelated sequence (GAPDH exon) was amplfied as further negative control. Primers are listed in Supplementary Table S2.

## Electronic supplementary material

Supplemental Figures

Supplemental Tables
