# Supplementary material for: Unravelling pathways downstream Sox6 induction in K562 erythroid cells by proteomic analysis
Source: Sci Rep. 2017 Oct 26;7:14088. doi: 10.1038/s41598-017-14336-6 (PMC5658338; doi:10.1038/s41598-017-14336-6)
Supplement: Supplementary file 1 — Supplemental Figures [file 41598_2017_14336_MOESM1_ESM.pdf]

**Unravelling pathways downstream Sox6 induction in K562 erythroid cells by proteomic analysis**

Gloria Barbarani, Antonella Ronchi, Margherita Ruoppolo, Lucia Santorelli, Robert Steinfelder, Sudharshan Elangovan, Cristina Fugazza, Marianna Caterino

**SUPPLEMENTAL FIGURES:**

Supplemental Figure 1

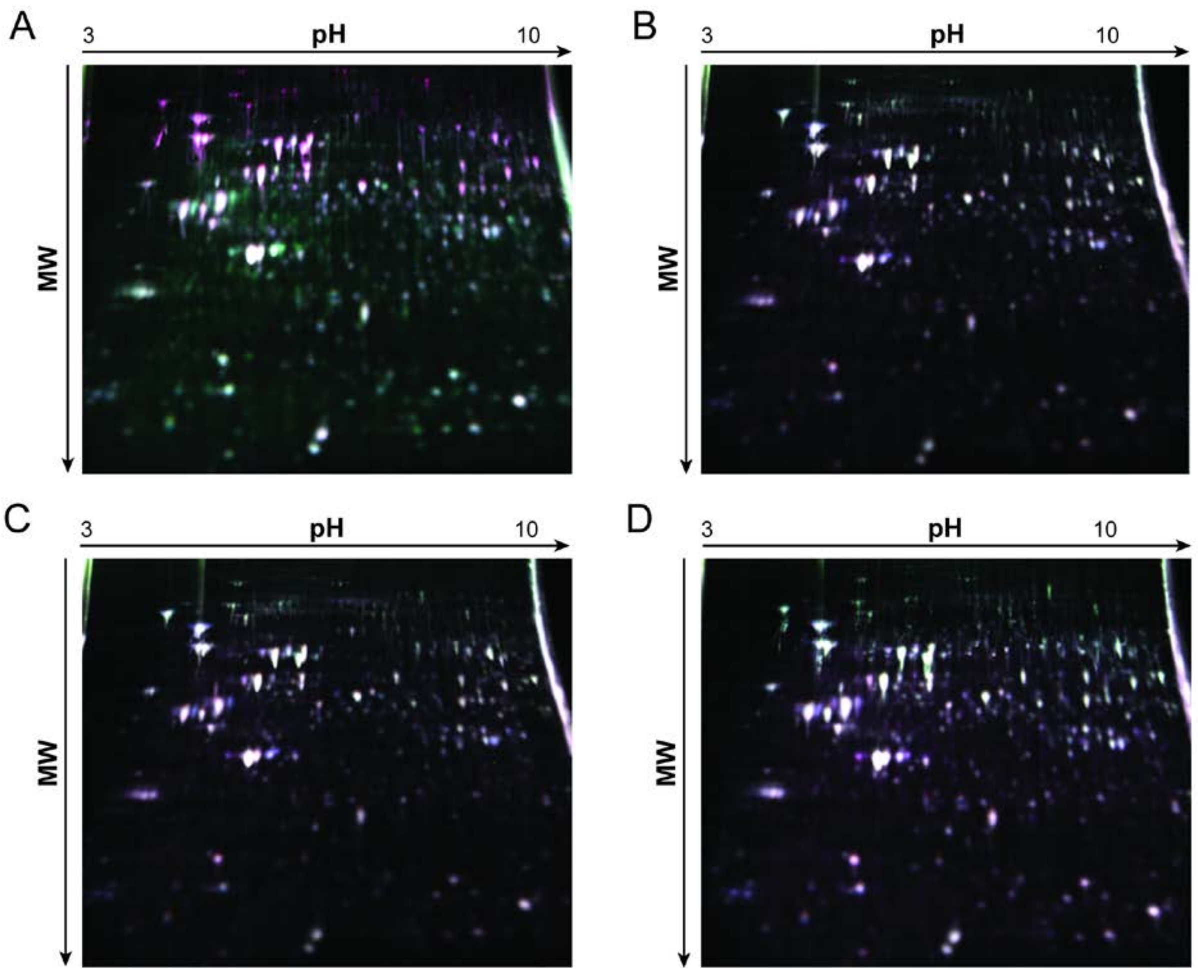


Supplemental Figure 1: DIGE analytical gel image overlays for the four biological replicates (gel A is the representative gel shown in Figure 2).

Supplemental Figure 2 page 1

*HSP90AB1*

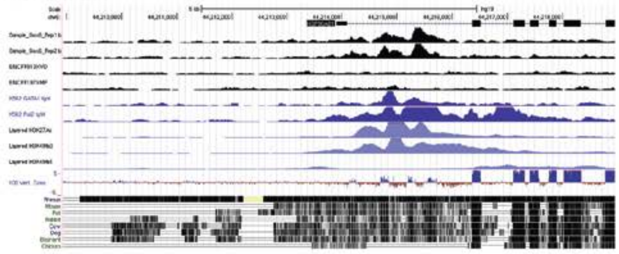

*EIF4H*

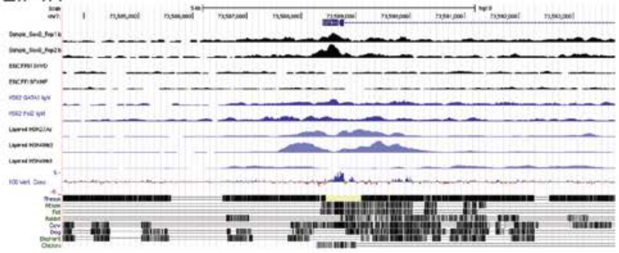

*TUBB*

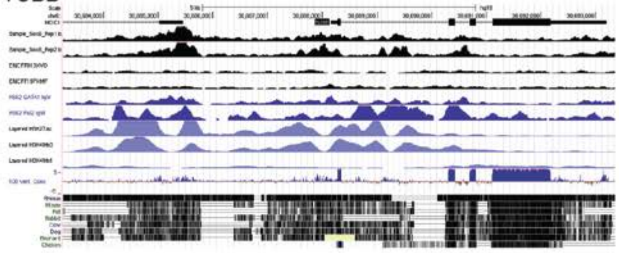

*HSPB1*

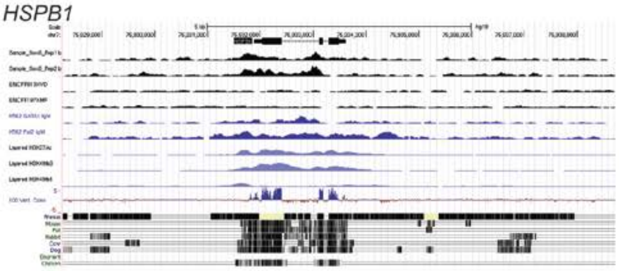

*ERP29*

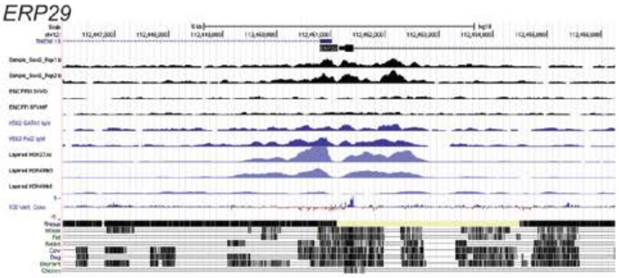

*ACTB*

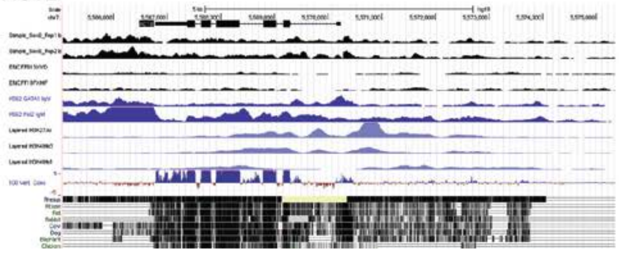

*RPLP0*

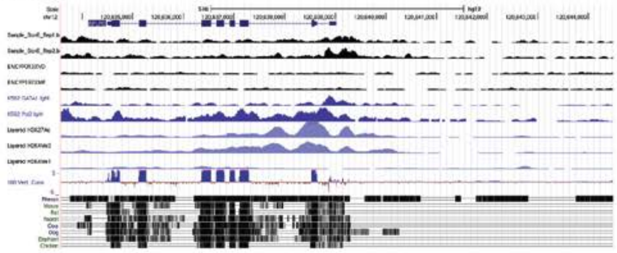

*GSS*

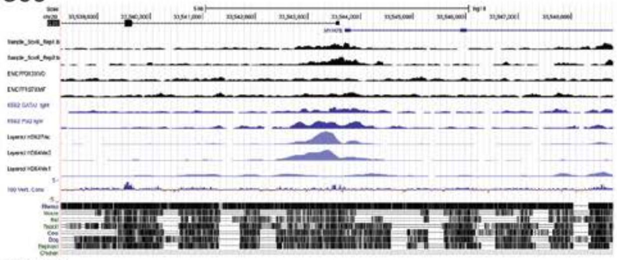

*GARS*

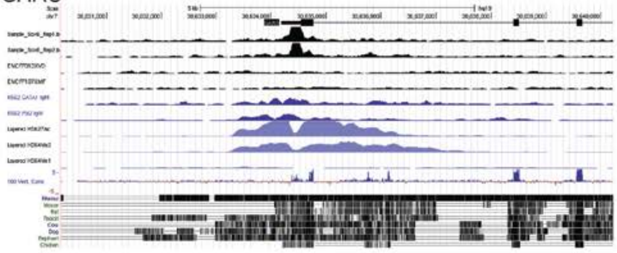

*HSPA4*

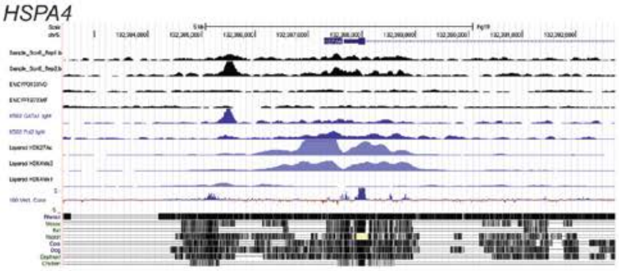

*EZR*

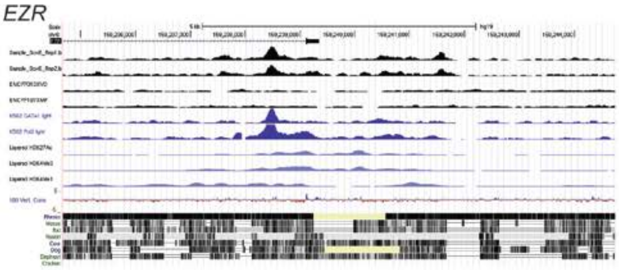

*CAPRIN1*

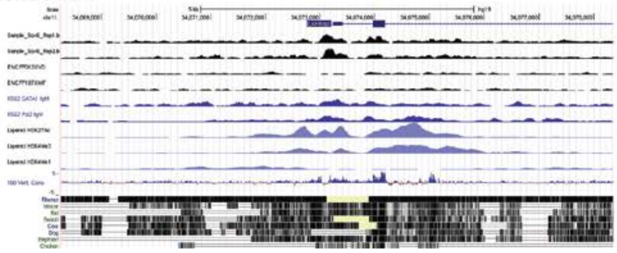

Supplemental Figure 2 page 2

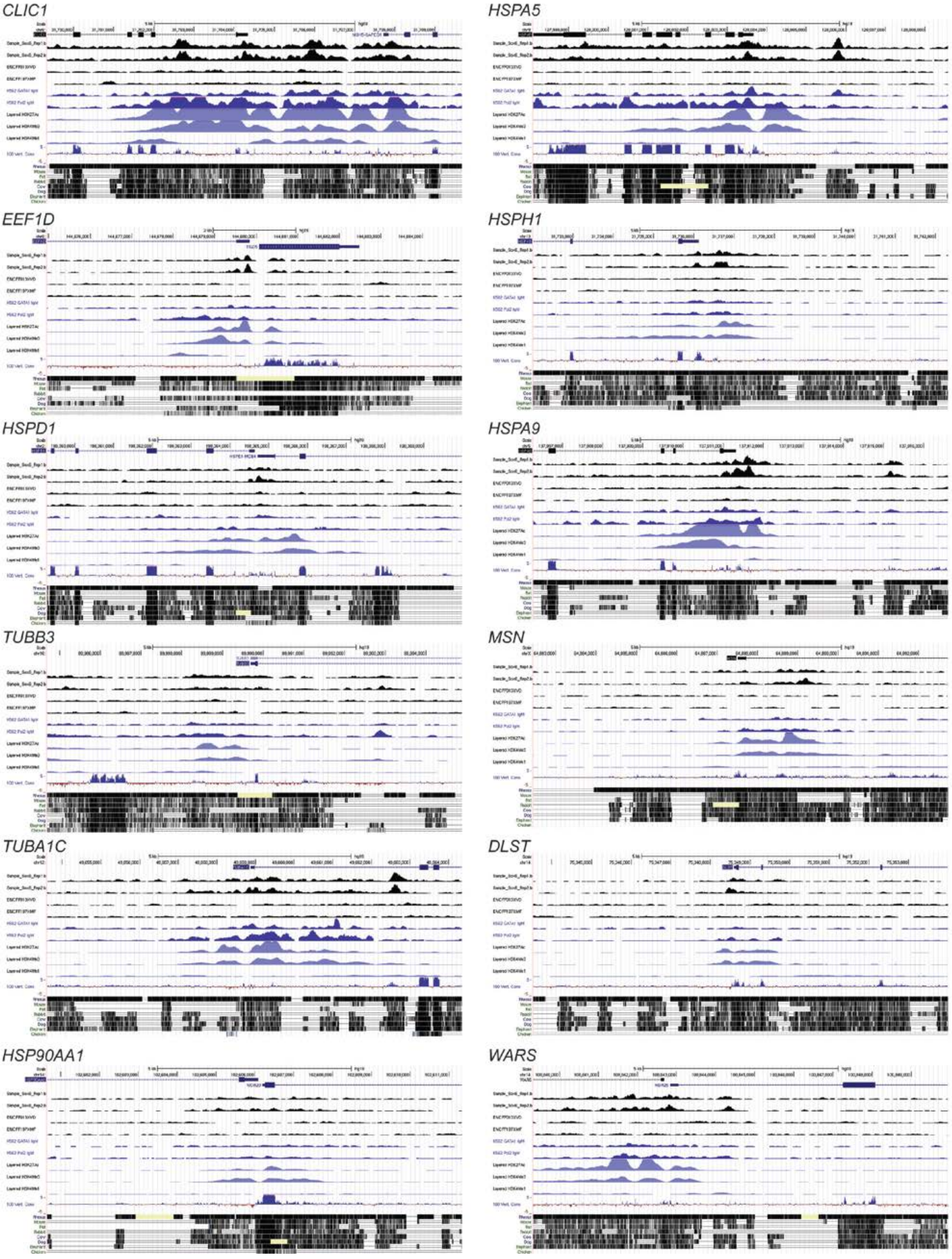

Supplemental Figure 2 page 3

*LMNA*

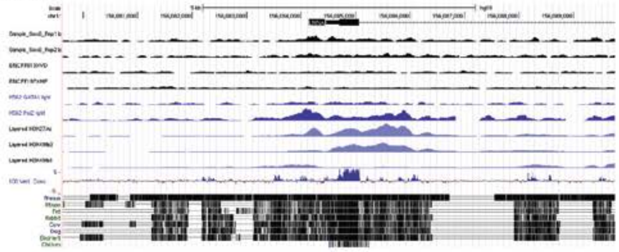

*HYOU1*

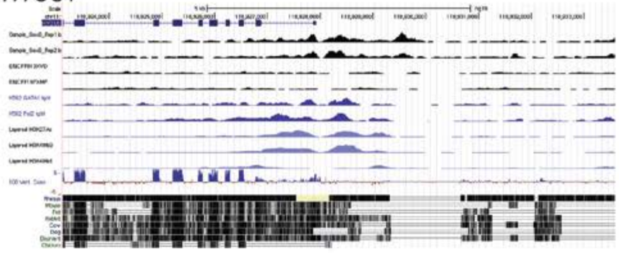

*VCP*

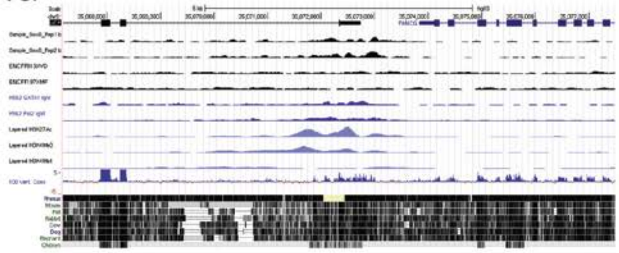

*MCM7*

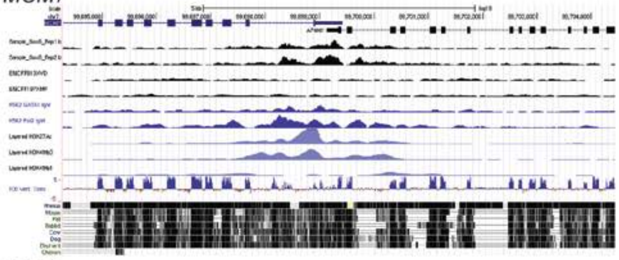

*AARS*

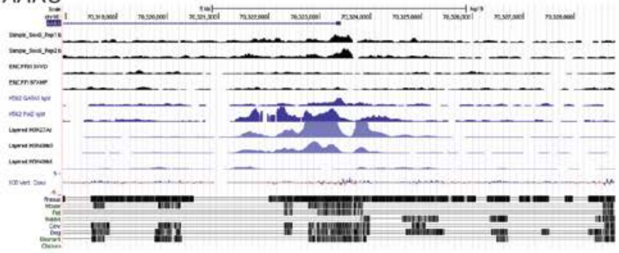

*PDCD6IP*

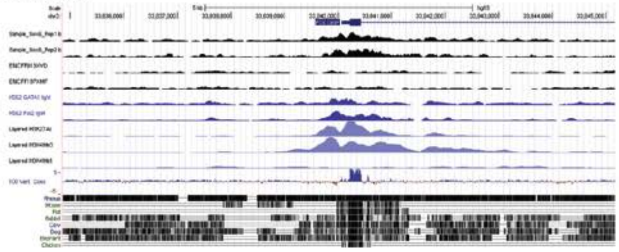

*IMPDH2*

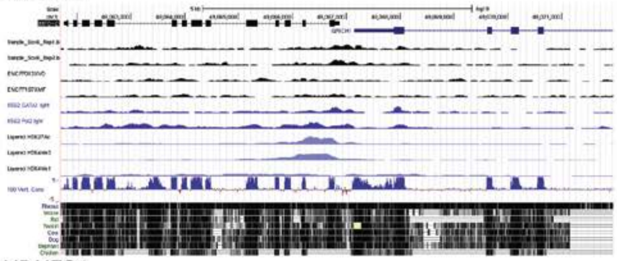

*NDUFS1*

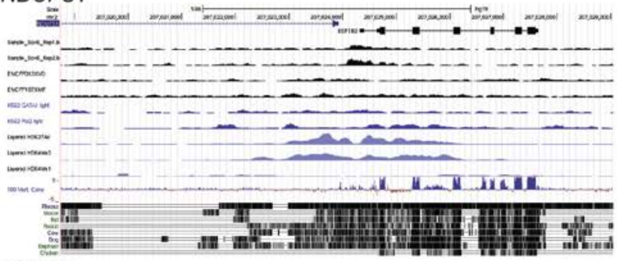

*HSPA8*

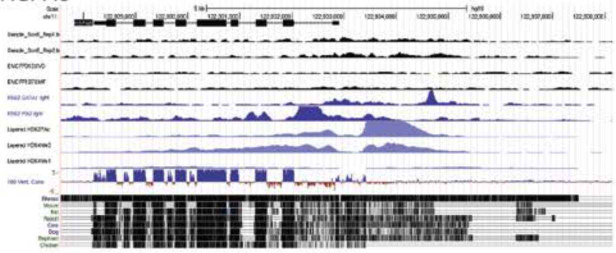

*CCT2*

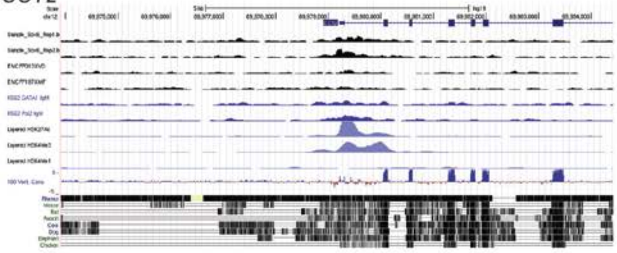

*UBA1*

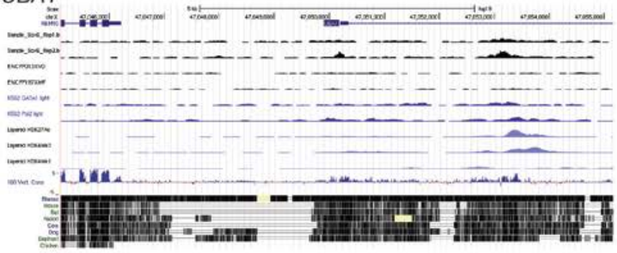

*TIMM44*

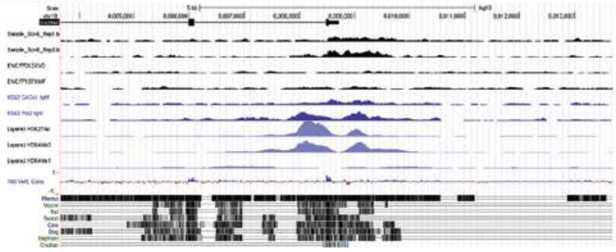

## Supplemental Figure 2 page 4

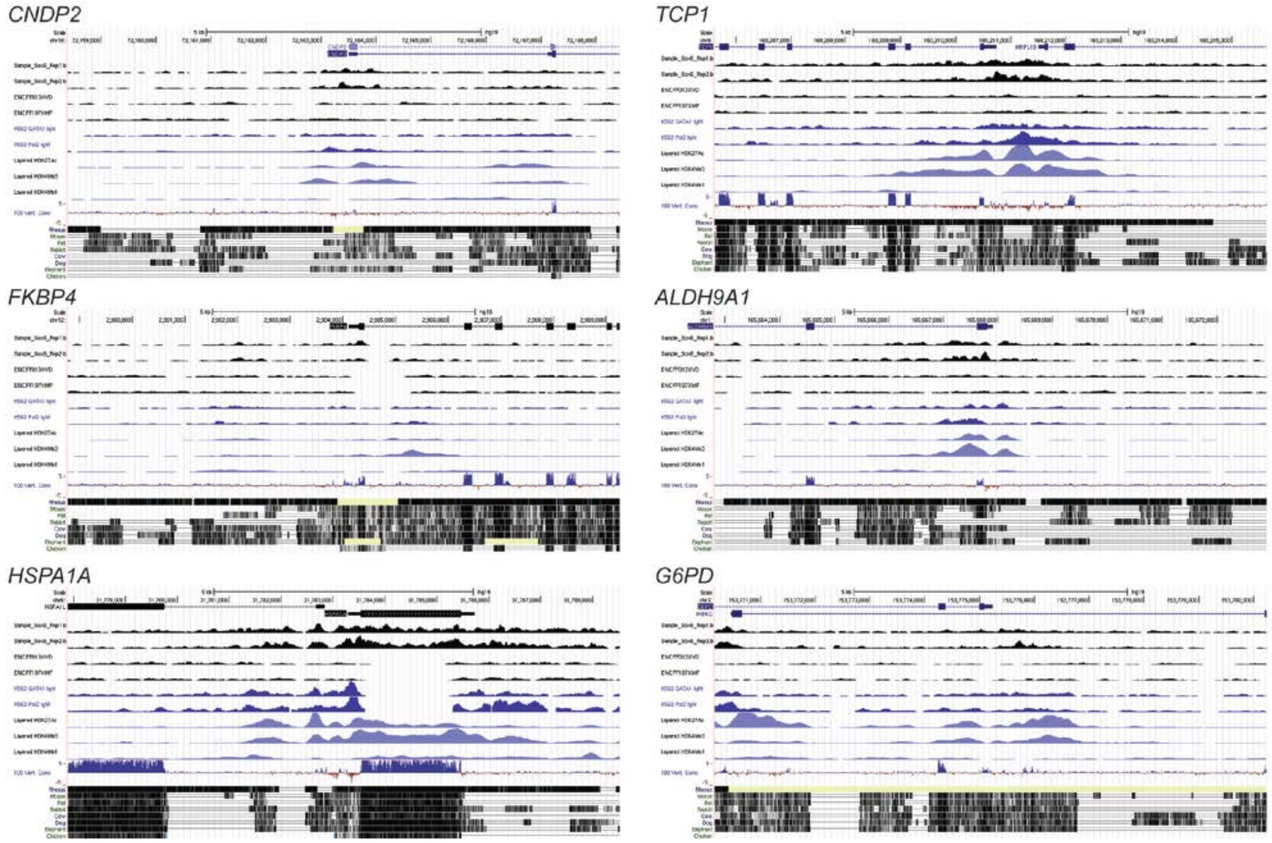


Supplemental Figure 2 (four pages):

Analysis of the regions spanning -/+5Kb around the transcriptional start site of the genes listed in Figure 4b.

In K562 cells, according to ENCODE ChIPseq datasets, all genes contain sites bound by both SOX6 and GATA1 within 1Kb. The comparison with human hepatocellular carcinoma HepG2 cells (expressing SOX6 but not GATA1) shows that 21/43 SOX6 peaks are K562-specific, 13/43 are common to both cell lines and 9/43 are HepG2-specific (within the same -/+5Kb regions, SOX6 peaks in K562 cells are present but map at different positions).

UCSC tracks are in the following order: two SOX6-ChIPseq replicates in K562 cells (ENCSR788RSW); two SOX6-ChIPseq replicates in HepG2 cells (ENCSR543BVU); GATA1-ChIPseq in K562 cells (ENCSR000EFT); POL2- (ENCSR000EHL), H3K27Ac- (ENCSR000AKP), H3K4me3- (ENCSR000AKU) and H3K4me1- (ENCSR000AKS) ChIPseq in K562 cells. Blocks in the bottom line correspond to the one hundred vertebrates base-wise conservation by PhyloP. Larger panels are available upon request.
