# Supplementary material for: Unravelling pathways downstream Sox6 induction in K562 erythroid cells by proteomic analysis
Source: Sci Rep. 2017 Oct 26;7:14088. doi: 10.1038/s41598-017-14336-6 (PMC5658338; doi:10.1038/s41598-017-14336-6)
Supplement: Supplementary file 2 — Supplemental Tables [file 41598_2017_14336_MOESM2_ESM.pdf]

Unravelling pathways downstream Sox6 induction in K562 erythroid cells by proteomic analysis

Gloria Barbarani, Antonella Ronchi, Margherita Ruoppolo, Lucia Santorelli, Robert Steinfelder, Sudharshan Elangovan, Cristina Fugazza, Marianna Caterino

Supplemental Table 1S. Details of mass spectometry identifications

# spot	mass/charge	Precursor	charge	Score	# peptide	istinct peptide sequence	Peptide sequence	overage (%)	(ppm)
2449	639.7100	1277.4054	2	49	3	3	R.TIAMDGTEGLVR.G + Oxidation (M)	6	156
	701.2100	1400.4054	2	74			R.IMNVIGEPIDER.G + Oxidation (M)		
	737.4200	1472.8254	2	14			K.TVLIMELINNVAK.A + Oxidation (M)		
1682	415.2200	828.4254	2	24	11	11	R.ALLFIPR.R		
	540.7000	1079.3854	2	23			R.APFDLFENK.K		
	576.1800	1150.3454	2	47			K.YIDQEELNK.T		
	597.7200	1193.4254	2	49			K.IDIIPNPQER.T		
	618.7400	1235.4654	2	42			R.RAPFDLFENK.K		
	621.7600	1241.5054	2	42			K.ADLINNLGTIAK.S		
	638.1800	1274.3454	2	60			R.ELISNASDALDK.I		
	656.0800	1310.1454	2	48			K.EDQTEYLEER.R		
	683.2800	1364.5454	2	78			R.TLTLVDTGIGMTK.A + Oxidation (M)		
	924.1600	1846.3054	2	79			R.NPDDITQEEYGEFYK.S		
	817.8600	2450.5582	3	11			R.YHTSQSGDEMTSLSEYVSRMK.E + Oxidation (M)		
1902	422.7100	843.4054	2	49	6	4	K.VGEVIVTK.D	7	
	617.2300	1232.4454	2	51			K.VGGTSDVEVNEK.K		
	575.1000	1722.2782	3	10			K.DPGMGAMGGMGGGMGGGMF.- + 3 Oxidation (M)		
	692.6300	2074.8682	3	11			K.EEKDPGMGAMGGMGGGMGGGMF.- + Oxidation (M)		
	692.9600	2075.8582	3	(9)			K.EEKDPGMGAMGGMGGGMGGGMF.- + Oxidation (M)		
	708.7100	2123.1082	3	(8)			K.EEKDPGMGAMGGMGGGMGGGMF.- + 4 Oxidation (M)		
2601	573.7300	1145.4454	2	47	2	2	K.LVFLGLDNAGK.T	11	
	438.8400	1313.4982	3	43			K.IDRPEAISEER.L		
2049	487.1500	972.2854	2	20	5	5	K.LVPVGYGIR.K	10	
	650.2000	1298.3854	2	72			R.GVVQELQQAISK.L		
	673.7000	1345.3854	2	67			K.VGTDLLEEEITK.F		
	679.7200	1357.4254	2	77			R.IASLEVENQSLR.G		
	771.6500	1541.2854	2	60			R.FYEQMNGPVAGASR.Q + Oxidation (M)		
2471	507.6900	1013.3654	2	23	3	3	R.DLTDYLMK.I + Oxidation (M)	12	
	566.6200	1131.2254	2	44			R.GYSFTTTAER.E		
	580.9000	1159.7854	2	37			K.KITALAPSTMK.I		
2420	416.2000	830.3854	2	26	8	8	R.VPFSLLR.G	37	206
	459.1300	916.2454	2	41			K.DGVVEITGK.H		
	471.1700	940.3254	2	29			R.AQLGGPEAAK.S		
	573.7200	1145.4254	2	37			K.TKDGVVEITGK.H		
	582.1400	1162.2654	2	74			R.LFDQAFGLPR.L		
	548.5300	1642.5682	3	35			R.AQLGGPEAAKSDETAAK.-		
	1038.5700	3112.6882	3	44			K.YTLPPGVDPTQVSSSLSPEGTLTVEAPMPK.L + Oxidation (M)		
	1081.5500	3241.6282	3	40			R.KYTLPPGVDPTQVSSSLSPEGTLTVEAPMPK.L + Oxidation (M)		
1962	466.1800	930.3454	2	42	3	2	K.GPSSVEDIK.A	10	204
	748.2000	2241.5782	3	(41)			K.MSVQPTVSLGGFEITPPVVLR.L + Oxidation (M)		
	1121.9100	2241.8054	2	57			K.MSVQPTVSLGGFEITPPVVLR.L + Oxidation (M)		
1892	443.8100	885.6054	2	16	5	5	R.RLSELLR.Y	5	138
	580.6900	1159.3654	2	43			K.SIYYITGESK.E		
	625.2200	1248.4254	2	46			K.EQVANSAFVER.V		
	625.2200	1248.4254	2	(32)			K.EQVANSAFVER.V		
	757.3100	1512.6054	2	73			R.GVVDSEDLPLNISR.E		
1892	618.2200	1234.4254	2	51	2	2	K.DQVANSAFVER.L	3	126
	757.3100	1512.6054	2	73			R.GVVDSEDLPLNISR.E		
1872	415.6400	829.2654	2	33	7	5	R.DLADVAAR.E	16	291
	515.1600	1028.3054	2	(40)			R.GVSAVVVGADR.V		
	515.2000	1028.3854	2	49			R.GVSAVVVGADR.V		
	525.6300	1049.2454	2	(50)			R.TALTTTISSR.D		
	525.6900	1049.3654	2	54			R.TALTTTISSR.D		
	581.6800	1161.3454	2	53			K.VGTYQLAIVAK.H		
	719.0800	1436.1454	2	53			R.GSLQILDQLLLPK.Q		
1872	508.1600	1014.3054	2	53	4	4	K.DVNAAIATIK.T	11	199
	543.2200	1084.4254	2	39			K.EIIDLVLDR.I		
	851.3600	1700.7054	2	56			R.AVFVDLEPTVIDEVR.T		
	1004.2300	2006.4454	2	10			K.TIGGGDDSFNTFFSETGAGK.H		
2140	565.6800	1129.3454	2	7	5	4	R.FPGQLNADLR.K	10	166
	580.2600	1158.5054	2	50			K.LAVNMVPFPR.L + Oxidation (M)		
	580.2600	1158.5054	2	(50)			K.LAVNMVPFPR.L + Oxidation (M)		
	668.2400	1334.4654	2	68			R.IMNTFSVVPSPK.V + Oxidation (M)		
	816.2500	1630.4854	2	37			R.AILVDLEPGTMDSVR.S + Oxidation (M)		
1567	415.1400	828.2654	2	23	10	6	R.ALLFIPR.R	7	387
	540.5500	1079.0854	2	23			R.APFDLFENK.K		
	580.6000	1159.1854	2	55			K.SIYYITGESK.E		
	618.5800	1235.1454	2	35			R.RAPFDLFENK.K		
	625.0500	1248.0854	2	(21)			K.EQVANSAFVER.V		
	625.0500	1248.0854	2	(18)			K.EQVANSAFVER.V		
	625.0500	1248.0854	2	(17)			K.EQVANSAFVER.V		
	625.0600	1248.1054	2	46			K.EQVANSAFVER.V		
	625.0700	1248.1254	2	(39)			K.EQVANSAFVER.V		
	757.1300	1512.2454	2	71			R.GVVDSEDLPLNISR.E		
1598	565.6100	1129.2054	2	11	5	4	R.FPGQLNADLR.K	9	283
	580.1300	1158.2454	2	43			K.LAVNMVPFPR.L + Oxidation (M)		
	668.2600	1334.5054	2	(59)			R.IMNTFSVVPSPK.V + Oxidation (M)		
	668.2600	1334.5054	2	63			R.IMNTFSVVPSPK.V + Oxidation (M)		
	731.5700	1461.1254	2	46			K.EVDEQMLNVQNK.N + Oxidation (M)		
1131	487.1600	972.3054	2	36	6	6	K.QALEDLER.T	10	285
	543.1900	1084.3654	2	52			R.LVSQGLEALR.S		
	573.1900	1144.3654	2	45			K.VAELLGESDGR.R		
	616.6800	1231.3454	2	42			R.LSQEEILGSTR.L		
	497.1600	1488.4582	3	17			R.SEQAVAQLEEEKK.H		
	764.1700	1526.3254	2	4			K.VPQARMSGLVLGQR.D + Oxidation (M)		
1526	471.1400	940.2654	2	53	3	3	R.ALAEGVLLR.T	5	286
	493.6300	985.2454	2	34			K.QQLEELAR.Q		
	514.6600	1027.3054	2	57			R.AIENELLAR.N		
2298	481.1000	960.1854	2	31	4	4	R.SAGLFQNPK.Q	21	324
	502.1300	1002.2454	2	16			R.FLQLTPER.C		
	716.1500	1430.2854	2	69			R.SLIIDEGEDDLGR.G		
	874.8100	2621.4082	3	63			K.GMGSGQLQNLGAAVAILGGPGTVQGVVR.F + Oxidation (M)		
2316	664.6000	1327.1854	2	50	2	2	K.NSNPALNDNLEK.G	14	361
	922.6400	1843.2654	2	66			K.LAALNPESNTAGLDIFAK.F		
2318	538.1700	1074.3254	2	29	5	3	K.QGQDNLSSVK.E	13	212
	567.7000	1133.3854	2	50			K.SLNILTAFQK.K		
	862.6900	1723.3654	2	61			K.ILDQGEDFPASEMTR.I + Oxidation (M)		
	575.5000	1723.4782	3	(43)			K.ILDQGEDFPASEMTR.I + Oxidation (M)		
	575.7300	1724.1682	3	(39)			K.ILDQGEDFPASEMTR.I + Oxidation (M)		
2318	531.1300	1060.2454	2	38	5	5	K.AAIISAEGDSK.A	14	258
	531.6200	1061.2254	2	42			R.QVSDDLTER.A		
	531.6400	1061.2654	2	(18)			R.QVSDDLTER.A		
	575.1100	1148.2054	2	50			R.FDAGELITQR.E		
	402.4800	1204.4182	3	25			R.FVVEKAEQQK.K		
2173	406.6500	811.2854	2	31	9	7	R.DVFLYR.A	25	266
	481.1500	960.2854	2	40			R.LVLQYAPSA.-		
	486.0900	970.1654	2	23			K.LDRLDLAR.K		
	509.1400	1016.2654	2	45			R.DSIVAELDR.E		

	768.6300	1535.2454	2	(32)			R.DSIVAELDREMSR.S + Oxidation (M)		
	512.8100	1535.4082	3	66			R.DSIVAELDREMSR.S + Oxidation (M)		
	699.5100	2095.5082	3	30			M.APPAPGPASGGSGEVDELFDVK.N		
	718.8800	2153.6182	3	(55)			K.FGVVLDEIKPSSAPELQAVR.M		
	718.8800	2153.6182	3	82			K.FGVVLDEIKPSSAPELQAVR.M		
2014	484.6100	967.2054	2	39	6	6	R.GNVGFVFTK.E	29	366
	609.0600	1216.1054	2	52			K.IIQLLDDYPK.C		
	657.1600	1312.3054	2	64			K.TSFFQALGITTK.I		
	714.6600	1427.3054	2	44			R.GTIEILSDVQLIK.T		
	948.1100	1894.2054	2	83			R.VLALSVETDYTFPLAEK.V		
	917.8800	2750.6182	3	42			K.AFLADPSAFVAAAPVAAATTAAPAAAAAPAK.V		
976	437.0800	872.1454	2	30	14	12	K.FADFMVK.D + Oxidation (M)	18	291
	457.1400	912.2654	2	24			R.IYLYLTK.V		
	459.5200	917.0254	2	16			K.ELSEALTR.H		
	474.5500	947.0854	2	18			R.LLEFNQGK.L		
	492.7000	983.3854	2	66			K.VGISPDKLR.F		
	492.7200	983.4254	2	(21)			K.VGISPDKLR.F		
	555.6200	1109.2254	2	38			K.GEFTIETEGK.T		
	555.6200	1109.2254	2	(34)			K.GEFTIETEGK.T		
	624.1600	1246.3054	2	59			K.TVNVVQFEPSK.G		
	634.6700	1267.3254	2	29			R.YPLFEGQETGK.K		
	696.1500	1390.2854	2	60			K.LPFAAAQIGNSFR.N		
	809.8400	1617.6654	2	17			R.SSMDGAGAEEVLAPLR.L + Oxidation (M)		
	543.0700	1626.1882	3	15			K.TSYGWIEIVGCADR.S		
	948.0200	2841.0382	3	12			K.TFIGPGGNMPGYLRPETAQGIFLNFK.R + Oxidation (M)		
980	408.6600	815.3054	2	38	22	14	K.VGMQIPR.F + Oxidation (M)	19	286
	431.1000	860.1854	2	29			R.LTEPMVR.N + Oxidation (M)		
	532.6600	1063.3054	2	(20)			R.FEAPLFNAR.I		
	532.6700	1063.3254	2	37			R.FEAPLFNAR.I		
	549.1100	1096.2054	2	40			K.SATYVNTEGR.A		
	578.1800	1154.3454	2	36			R.LEEVSPNLVR.Y		
	591.0400	1180.0654	2	(53)			R.VAGMLQSFQGK.D + Oxidation (M)		
	591.1100	1180.2054	2	(58)			R.VAGMLQSFQGK.D + Oxidation (M)		
	591.1400	1180.2654	2	(66)			R.VAGMLQSFQGK.D + Oxidation (M)		
	591.1500	1180.2854	2	68			R.VAGMLQSFQGK.D + Oxidation (M)		
	625.5100	1249.0054	2	42			K.DFYMTDSISR.A + Oxidation (M)		
	685.5400	1369.0654	2	(27)			R.GNDMQVGTYIEK.M + Oxidation (M)		
	685.5900	1369.1654	2	33			R.GNDMQVGTYIEK.M + Oxidation (M)		
	685.6900	1369.3654	2	(18)			R.GNDMQVGTYIEK.M + Oxidation (M)		
	467.8500	1400.5282	3	39			K.KPMVVLGSSALQR.N + Oxidation (M)		
	475.8100	1424.4082	3	(32)			R.NRLEEVSPNLVR.Y		
	475.8500	1424.5282	3	36			R.NRLEEVSPNLVR.Y		
	764.7400	1527.4654	2	64			R.NDGAAILAAVSSIAQK.I		
	510.4700	1528.3882	3	(15)			R.NDGAAILAAVSSIAQK.I		
	804.7200	1607.4254	2	69			R.FASEIAGVDDLGTTGR.G		
	1054.6700	2107.3254	2	(40)			R.ALSEIAGMTLPYDTLDQVR.N + Oxidation (M)		
	703.5400	2107.5982	3	40			R.ALSEIAGMTLPYDTLDQVR.N + Oxidation (M)		
1080	431.6000	861.1854	2	(20)	34	20	R.ETGVDLTK.D	34	313
	431.6000	861.1854	2	21			R.ETGVDLTK.D		
	459.0900	916.1654	2	(10)			K.LFEMAYK.K + Oxidation (M)		
	459.1300	916.2454	2	13			K.LFEMAYK.K + Oxidation (M)		
	479.5700	957.1254	2	20			K.VLENAEGAR.T		
	616.0600	1230.1054	2	66			R.QAASSLQQASLK.L		
	621.6700	1241.3254	2	65			K.DAGQISGLNVLR.V		
	621.6900	1241.3654	2	(48)			K.DAGQISGLNVLR.V		
	645.7000	1289.3854	2	79			K.VQQTVQDLFGR.A		
	681.2000	1360.3854	2	92			R.AQFEGIVTDLIR.R		
	454.4700	1360.3882	3	(51)			R.AQFEGIVTDLIR.R		
	725.6200	1449.2254	2	53			R.TTPSVVAFTADGER.L		
	725.6600	1449.3054	2	(52)			R.TTPSVVAFTADGER.L		
	484.1700	1449.4882	3	(22)			R.TTPSVVAFTADGER.L		
	731.6500	1461.2854	2	60			K.SDIGEVILVGGMTR.M + Oxidation (M)		
	737.1500	1472.2854	2	63			R.EQQIVIQSSGGLSK.D		
	737.1500	1472.2854	2	(61)			R.EQQIVIQSSGGLSK.D		
	784.6300	1567.2454	2	75			R.QAVTNPNNTFYATK.R		
	523.7100	1568.1082	3	37			K.LYSPSQIGAFVLMK.M + Oxidation (M)		
	785.2300	1568.4454	2	(23)			K.LYSPSQIGAFVLMK.M + Oxidation (M)		
	531.5000	1591.4782	3	(37)			K.LLGQFTLIGIPPAPR.G		
	796.7500	1591.4854	2	40			K.LLGQFTLIGIPPAPR.G		
	796.7900	1591.5654	2	(36)			K.LLGQFTLIGIPPAPR.G		
	823.1600	1644.3054	2	98			R.VINEPTAAALAYGLDK.S		
	847.6200	1693.2254	2	41			K.NAVITVPAYFNDSQR.Q		
	569.3600	1705.0582	3	(42)			R.ETGVDLTKDNMALQR.V + Oxidation (M)		
	569.3600	1705.0582	3	44			R.ETGVDLTKDNMALQR.V + Oxidation (M)		
	569.4300	1705.2682	3	(24)			R.ETGVDLTKDNMALQR.V + Oxidation (M)		
	853.7100	1705.4054	2	(24)			R.ETGVDLTKDNMALQR.V + Oxidation (M)		
	1028.6900	2055.3654	2	94			K.STNGDTFLGGEDFDQALLR.H		
	686.1300	2055.3682	3	(43)			K.STNGDTFLGGEDFDQALLR.H		
	702.0800	2103.2182	3	29			R.VINEPTAAALAYGLDKSEDK.V		
	811.8800	2432.6182	3	45			R.EQQIVIQSSGGLSKDDIENMVK.N + Oxidation (M)		
	813.1800	2436.5182	3	55			K.AMQDAEVSKSDIGEVILVGGMTR.M + 2 Oxidation (M)		
1080	402.6100	803.2054	2	24	25	19	K.ITITNDK.G	33	285
	495.1500	988.2854	2	41			R.LSKEDIER.M		
	541.1100	1080.2054	2	28			K.LLQDFFNGK.E		
	590.6300	1179.2454	2	31			K.VQVEYKGETK.S		
	600.1800	1198.3454	2	(20)			K.DAGTIAGLNVLR.I		
	600.2300	1198.4454	2	42			K.DAGTIAGLNVLR.I		
	407.7200	1220.1382	3	33			R.AMTKDNNLLGK.F + Oxidation (M)		
	614.6300	1227.2454	2	58			K.VEIIANDQGNR.T		
	615.0100	1228.0054	2	(4)			K.VEIIANDQGNR.T		
	627.1200	1252.2254	2	48			R.FEELNADLFR.G		
	635.5000	1268.9854	2	53			R.FDDAVVQSDMK.H + Oxidation (M)		
	660.1000	1318.1854	2	72			K.NSLESYAFNMK.A + Oxidation (M)		
	476.1200	1425.3382	3	44			R.RFDDAVVQSDMK.H + Oxidation (M)		
	494.1600	1479.4582	3	25			R.ARFEELNADLFR.G		
	744.1700	1486.3254	2	77			R.TTPSYVAFTDTER.L		
	817.1200	1632.2254	2	56			K.SFYPEEVSSMVLTK.M + Oxidation (M)		
	830.1900	1658.3654	2	66			R.IINEPTAAAIAYGLDK.K		
	555.7300	1664.1682	2	(44)			K.NQVAMNPTNTVFDAK.R + Oxidation (M)		
	833.3300	1664.6454	2	76			K.NQVAMNPTNTVFDAK.R + Oxidation (M)		
	833.5300	1665.0454	2	(18)			K.NQVAMNPTNTVFDAK.R + Oxidation (M)		
	833.6400	1665.2654	2	(45)			K.NQVAMNPTNTVFDAK.R + Oxidation (M)		
	564.4100	1690.2082	3	14			K.STAGDTHLGGEDFDNR.M		
	991.1800	1980.3454	2	(27)			K.TVTNAVVTVPAYFNDSQR.Q		
	661.4800	1981.4182	3	44			K.TVTNAVVTVPAYFNDSQR.Q		
	1130.6600	2259.3054	2	79			K.SINPDEAVAYGAAVQAAILSGDK.S		
1273	486.5600	971.1054	2	39	20	13	K.SSLGPVGLDK.M	29	312
	486.5800	971.1454	2	(22)			K.SSLGPVGLDK.M		
	573.6800	1145.3454	2	61			R.YPVNSVNILK.A		
	574.2000	1146.3854	2	35			R.EQLAIAEFAR.S		
	594.6700	1187.3254	2	22			K.QAGVFEPTIVK.V		

	603.1900	1204.3654	2	(52)			K.FATEAAITILR.I		
	603.1900	1204.3654	2	57			K.FATEAAITILR.I		
	615.0900	1228.1654	2	45			K.YFVEAGAMAVR.R + Oxidation (M)		
	616.1600	1230.3054	2	38			R.ICDDELILIK.N		
	511.4600	1531.3582	3	(61)			R.SQNVMAAASIANIVK.S + Oxidation (M)		
	766.7200	1531.4254	2	94			R.SQNVMAAASIANIVK.S + Oxidation (M)		
	773.7500	1545.4854	2	26			R.DNKQAGVFEPTIVK.V		
	881.6700	1761.3254	2	67			R.YINENLIVNTDELGR.D		
	882.1600	1762.3054	2	(30)			R.YINENLIVNTDELGR.D		
	641.5100	1921.5082	3	(67)			K.LGVQVVITDPEKLDQIR.Q		
	641.5100	1921.5082	3	72			K.LGVQVVITDPEKLDQIR.Q		
	1060.1800	2118.3454	2	99			K.MLVDDIGDVTITNDGATILK.L + Oxidation (M)		
	707.1700	2118.4882	3	(68)			K.MLVDDIGDVTITNDGATILK.L + Oxidation (M)		
	1176.7500	2351.4854	2	(27)			R.SLLVIPNTLAVNAAQDSTDLVAK.L		
	1176.7600	2351.5054	2	53			R.SLLVIPNTLAVNAAQDSTDLVAK.L		
1376	503.1300	1004.2454	2	28	11	11	K.FLDQNLQK.Y	5	295
	565.1900	1128.3654	2	61			K.IEVLEEELR.L		
	570.1700	1138.3254	2	48			R.AELIVQPELK.Y		
	628.0500	1254.0854	2	57			K.AITGFDDPFSGK.T		
	511.0800	1530.2182	3	29			R.LKNTLTQTTENLR.R		
	769.7800	1537.5454	2	81			R.LLEAQIATGGIIDPK.E		
	793.7500	1585.4854	2	61			R.ALLQAILQTEDMLK.V		
	802.1600	1602.3054	2	26			K.SVEEVASEIQPFLR.G		
	708.1200	2121.3382	3	24			R.TMIQSPSGVILQEAADVHAR.Y		
	811.8500	2432.5282	3	23			K.AQQIHSQTSQQYPLYDLDLGK.F		
	1040.6200	3118.8382	3	51			K.IGLVRPGTALELLEAQAATGFIVDPVSNLR.L		
1438	539.0300	1076.0454	2	36	5	5	K.LMPMQQIAN.- + 2 Oxidation (M)	13	380
	550.5500	1099.0854	2	37			K.DGSVAIASKPR.E		
	628.0800	1254.1454	2	67			K.GMGGAMDLVSSAK.T + 2 Oxidation (M)		
	817.1100	1632.2054	2	88			R.MVSSYVGENAEFER.Q + Oxidation (M)		
	807.4400	2419.2982	3	38			K.ETVTILPGASFFSSDESFAMIR.G + Oxidation (M)		
1438	565.5900	1129.1654	2	54	3	3	K.VTADVINAAEK.L	7	393
	673.1300	1344.2454	2	71			K.GTIQVITQGTSLK.N		
	744.5600	1487.1054	2	61			R.AGTGVDNVDLEAATR.K		
1456	438.6300	875.2454	2	28	18	14	K.DEIDSAVK.X	20	313
	472.0700	942.1254	2	20			R.ETRDVAPR.I		
	500.6300	999.2454	2	(42)			K.IDKELINR.I		
	500.6300	999.2454	2	44			K.IDKELINR.I		
	596.1800	1190.3454	2	49			K.GIDYDKLIVR.F		
	701.1200	1400.2254	2	63			K.DYTSGAMLTGELK.K + Oxidation (M)		
	702.6200	1403.2254	2	62			K.AGNASKDEIDSAVK.X		
	468.8900	1403.6482	3	(48)			K.AGNASKDEIDSAVK.X		
	727.9900	1453.9654	2	82			R.DFNQVLDAYENK.K		
	765.0900	1528.1654	2	62			K.DYTSGAMLTGELKK.A + Oxidation (M)		
	516.1600	1545.4582	3	6			R.KDYTSGAYLTGELK.K		
	855.1100	1708.2054	2	22			K.EVTDEIVKEFMTPR.K + Oxidation (M)		
	863.1900	1724.3654	2	22			K.EVTDEIVKEFYTPR.K		
	600.8500	1799.5282	3	(27)			K.MSASDPNSSIFLTDTAK.Q + Oxidation (M)		
	900.8700	1799.7254	2	90			K.MSASDPNSSIFLTDTAK.Q + Oxidation (M)		
	901.1100	1800.2054	2	51			K.FSASDPNSSIFLTDTAK.Q		
	908.6200	1815.2254	2	82			K.YSASDPNSSIFLTDTAK.Q		
	606.0900	1815.2482	3	(16)			K.YSASDPNSSIFLTDTAK.Q		
1500	424.6700	847.3254	2	29	10	7	K.AFEPATGR.V	13	298
	543.1200	1084.2254	2	(39)			K.EVNLAVQNAK.A		
	543.1500	1084.2854	2	43			K.EVNLAVQNAK.A		
	564.6500	1127.2854	3	83			K.IGDPLLEDTR.M		
	613.5700	1225.1254	2	27			K.VSFTGSVPTGMK.I + Oxidation (M)		
	685.1900	1368.3654	2	21			R.IKIGDPLLEDTR.M		
	770.6700	1539.3254	2	76			R.ANDTTFGLAAGVFTR.D		
	514.1600	1539.4582	3	(57)			R.ANDTTFGLAAGVFTR.D		
	771.1700	1540.3254	2	(69)			R.ANDTTFGLAAGVFTR.D		
	644.7500	1931.2282	3	25			R.VEPADASGTEKAFEPATGR.V		
1500	415.2000	828.3854	2	24	13	9	R.ALLFIPR.R	13	339
	443.6300	885.2454	2	28			R.RLSELLR.Y		
	576.0900	1150.1654	2	47			K.YIDQEELNK.T		
	580.6200	1159.2254	2	48			K.SIYYITGESK.E		
	618.5600	1235.1054	2	38			R.RAPFDLFENK.K		
	625.0500	1248.0854	2	(45)			K.EQVANSAFVER.V		
	625.1200	1248.2254	2	(44)			K.EQVANSAFVER.V		
	625.5700	1249.1254	2	46			K.EQVANSAFVER.V		
	505.1100	1512.3082	3	(49)			R.GVVDSEDLPLNISR.E		
	757.2000	1512.3854	2	67			R.GVVDSEDLPLNISR.E		
	612.3700	1834.0882	3	7			K.AQALRDNSTMGYMMAK.K + 3 Oxidation (M)		
	616.3800	1846.1182	3	(26)			R.NPDDITQEEYGEFYK.S		
	924.1300	1846.2454	2	65			R.NPDDITQEEYGEFYK.S		
1500	596.7000	1191.3854	2	31	3	3	K.AKPAEAPAAAAPK.A	8	265
	712.6200	1423.2254	2	84			R.NVEAMNFADIER.T + Oxidation (M)		
	739.6600	1477.3054	2	53			K.TPAFAESVTEGDVR.W		
1500	471.1600	940.3054	2	52	3	3	R.ALAEGVLLR.T	5	343
	493.5600	985.1054	2	21			K.QQLEELAR.Q		
	514.6300	1027.2454	2	43			R.AIENELLAR.N		
1770	459.0900	916.1654	2	35	6	6	K.LGELTGTVK.E	14	344
	467.6000	933.1854	2	25			K.TIESETVR.T		
	695.1200	1388.2254	2	50			R.LLDISASSTEQIL.-		
	716.6500	1431.2854	2	64			R.ILDIDNVDLAMGK.M + Oxidation (M)		
	719.0800	1436.1454	2	84			K.TEMSEVLTEILR.V + Oxidation (M)		
	482.0700	1443.1882	3	42			R.RLEESDVLQEAR.R		
1770	475.1400	948.2654	2	40	10	7	R.LEAFLTQK.A	18	312
	492.1600	982.3054	2	35			R.LNQPGTPTR.T		
	499.0900	996.1654	2	16			R.YPIPPPDAK.E		
	499.1000	996.1854	2	(14)			R.YPIPPPDAK.E		
	527.6100	1053.2054	2	(32)			K.RIPEEILGK.V		
	527.6500	1053.2854	2	56			K.RIPEEILGK.V		
	651.1100	1300.2054	2	33			R.ISELGAGNGGVVTK.V		
	441.4200	1321.2382	3	44			K.VGELKDDDFER.I		
	661.6600	1321.3054	2	(20)			K.VGELKDDDFER.I		
	671.2000	1340.3854	2	11			R.DVKPSNILVNSR.G		
1382	410.6700	819.3254	2	30	7	6	K.SNILVFK.L	13	323
	424.1200	846.2254	2	30			R.AAGFNVLR.L		
	510.1500	1018.2854	2	38			R.DILAVLTMK.R + Oxidation (M)		
	523.5800	1045.1454	2	45			K.FVNPEDVAR.L		
	523.6000	1045.1854	2	(36)			K.FVNPEDVAR.L		
	751.6300	1501.2454	2	28			R.FTVLFPSGTPLPAR.R		
	1030.1500	2058.2854	2	65			R.VTPAVVAYSENEEIVGLAAK.Q		
1400	407.1100	812.2054	2	31	14	12	K.FSFDLGK.G	29	293
	420.1000	838.1854	2	24			K.FDSSLDR.K		
	432.1100	862.2054	2	24			K.QALLQYK.K		
	459.1400	916.2654	2	36			K.LEQSTIVK.E		
	530.0300	1058.0454	2	37			K.LYANMFER.L + Oxidation (M)		
	566.6100	1131.2054	2	48			K.ALELDSNNEK.G		
	578.6400	1155.2654	2	21			K.FQIPPNAELK.Y		

	578.7100	1155.4054	2	(21)			K.FQIPPNAELK.Y		
	704.6700	1407.3254	2	46			K.SNTAGSQSQVETEA.-		
	713.1000	1424.1854	2	26			R.EGTGTEMPMIGDR.V + 2 Oxidation (M)		
	766.6000	1531.1854	2	64			K.GEDLTEEEDGGIIR.R		
	975.6500	1949.2854	2	54			R.FEIGEGENLDLPYGLER.A		
	1050.7000	2099.3854	2	(40)			K.ATESGAQSAPLPMEGVDISPK.Q + Oxidation (M)		
	700.8200	2099.4382	3	54			K.ATESGAQSAPLPMEGVDISPK.Q + Oxidation (M)		
1506	618.6100	1235.2054	2	(30)	6	5	R.RAPFDLFENK.K	7	314
	618.6200	1235.2254	2	41			R.RAPFDLFENK.K		
	625.1000	1248.1854	2	48			K.EQVANSAFVER.V		
	674.5800	1347.1454	2	7			K.HFSVEGQLEFR.A		
	470.1900	1407.5482	3	8			K.EKYIDQEELNK.T		
	757.1600	1512.3054	2	75			R.GVVDSEDLPLNISR.E		
1506	548.5800	1095.1454	2	30	6	6	R.MMEVAAADVK.Q + 2 Oxidation (M)	16	301
	617.1500	1232.2854	2	52			K.TVFGVEPDLTR.E		
	843.7300	1685.4454	2	19			K.LPDGSEIPLPPILLGR.L		
	867.6600	1733.3054	2	37			R.EGGSIPVTLTFQEATGK.N		
	648.2000	1941.5782	3	23			K.QKLPDGSEIPLPPILLGR.L		
	740.8500	2219.5282	3	31			R.LVPNMTPEVVGEQVTSYLTK.K + Oxidation (M)		
631	620.1200	1238.2254	2	46	7	7	R.AFSDPFVEAEK.S	20	287
	432.4600	1294.3582	2	42			K.QDLPALEEKPR.N		
	661.1800	1320.3454	2	81			K.VLATAFDTTLGGR.K		
	748.0600	1494.1054	2	45			R.AGGIETIANEYSDR.C		
	760.1600	1518.3054	2	55			K.ELSTTLNADEAVTR.G		
	799.6600	1597.3054	2	108			R.NFTTEQVTAMLLSK.L + Oxidation (M)		
	635.1100	1902.3082	3	37			K.SNLAYDIVQLPTGLTGIK.V		
601	407.1600	812.3054	2	26	13	11	K.NIILGGVK.A	13	278
	414.6400	827.2654	2	17			K.ATLPSPDK.L		
	492.6400	983.2654	2	42			R.DEFEGLFK.Q		
	625.5700	1249.1254	2	39			R.AENYDIPSADR.H		
	652.6400	1303.2654	2	45			R.LDQPMTEIVSR.V + Oxidation (M)		
	808.7100	1615.4054	2	62			K.QPAENVNQYLTDPK.F		
	541.8300	1622.4682	3	(54)			R.LAGTQPLEVLEAVQR.S		
	812.2800	1622.5454	2	74			R.LAGTQPLEVLEAVQR.S		
	812.2800	1622.5454	2	(16)			R.LAGTQPLEVLEAVQR.S		
	904.6600	1807.3054	2	53			R.ALPAVQQNNLDEDLIR.K		
	918.7100	1835.4054	2	9			R.FEVQGLQPNGEEMTLK.Q + Oxidation (M)		
	942.1700	1882.3254	2	52			R.NEEDAAELVALAQAVNAR.A		
	954.6700	1907.3254	2	42			K.SLVASLAEPDFVVTDFAK.F		
601	450.6200	899.2254	2	36	8	8	R.LVSVLQNK.M	9	316
	473.6100	945.2054	2	14			R.SVLGEADQK.G		
	530.5500	1059.0854	2	50			R.GLEVTDDSPK.Y		
	543.6900	1085.3654	2	43			R.IVAVTGAEAQK.A		
	547.6300	1093.2454	2	47			K.SIDTGMGLER.L + Oxidation (M)		
	738.6600	1475.3054	2	16			K.DIINEEEVQFLK.T		
	756.1000	1510.1854	2	56			K.GLVVDMDGFEEER.K + Oxidation (M)		
	572.1800	1713.5182	3	44			R.TITVALADGGRPDNTGR.G		
745	410.1600	818.3054	2	31	19	14	K.GDIFLVR.G	17	302
	474.1600	946.3054	2	46			R.KGDIFLVR.G		
	481.5300	961.0454	2	(26)			R.ESIESEIR.R		
	481.6200	961.2254	2	28			R.ESIESEIR.R		
	516.6400	1031.2654	2	47			K.GDDLSTAILK.Q		
	525.1500	1048.2854	2	49			K.DVDLEFLAK.M		
	534.1400	1066.2654	2	45			K.MDELQLFR.G + Oxidation (M)		
	538.1500	1074.2854	2	51			K.LAGESESNLR.K		
	559.6200	1117.2254	2	15			R.ESIESEIRR.E		
	586.6600	1171.3054	2	34			R.GILLYGPPGTGK.T		
	621.6500	1241.2854	2	30			R.EVDIGIPDATGR.L		
	723.6200	1445.2254	2	(57)			R.IVSQLLTLMDGLK.Q + Oxidation (M)		
	723.7000	1445.3854	2	76			R.IVSQLLTLMDGLK.Q + Oxidation (M)		
	482.8100	1445.4082	3	(13)			R.IVSQLLTLMDGLK.Q + Oxidation (M)		
	778.7000	1555.3854	2	50			R.LDQLIYIPLPDEK.S		
	856.1000	1710.1854	2	51			R.VINQILTEMDGMSTK.K + 2 Oxidation (M)		
	1086.1000	2170.1854	2	(33)			R.QTNPSAMEVEEDDPVPEIR.R + Oxidation (M)		
	724.4200	2170.2382	3	55			R.QTNPSAMEVEEDDPVPEIR.R + Oxidation (M)		
	724.5000	2170.4782	3	(40)			R.QTNPSAMEVEEDDPVPEIR.R + Oxidation (M)		
1435	499.6100	997.2054	2	27	11	10	K.NIGVDNPAAK.V	25	297
	557.6200	1113.2254	2	29			K.EAVAMESYAK.A + Oxidation (M)		
	646.1100	1290.2054	2	(59)			K.LIEEVMIGEDK.L + Oxidation (M)		
	646.1500	1290.2854	2	66			K.LIEEVMIGEDK.L + Oxidation (M)		
	654.1500	1306.2854	2	59			K.ILIANTGMDTDK.I + Oxidation (M)		
	665.6400	1329.2654	2	72			R.GATQQILDEAER.S		
	747.6500	1493.2854	2	56			R.QDLMNIAGTTLSSK.L + Oxidation (M)		
	759.1900	1516.3654	2	55			R.LTSFIGAIAIGDLVK.S		
	782.6800	1563.3454	2	57			R.DASLMVTNDGATILK.N + Oxidation (M)		
	956.8500	1911.6854	2	57			K.LGGSLADSYLDEGFLLDK.K		
	788.5700	2362.6882	3	30			R.MLPTIIADNAGYDSADLVAQLR.A + Oxidation (M)		
1013	459.5900	917.1654	2	21	13	12	K.VLEDSDLK.K	23	314
	493.6000	985.1854	2	22			R.LTPEEIER.M		
	614.6000	1227.1854	2	52			R.VEIIANDQGNR.I		
	617.0800	1232.1454	2	46			K.DAGTIAGLNVMR.I + Oxidation (M)		
	665.1000	1328.1854	2	55			K.FEELNMDLFR.S + Oxidation (M)		
	699.1800	1396.3454	2	47			K.ELEEIVQPIISK.L		
	730.6800	1459.3454	2	50			K.SDIDEIVLVGGSTR.I		
	776.7400	1551.4654	2	69			K.TFAPEEISAMVLTK.M + Oxidation (M)		
	776.7400	1551.4654	2	(68)			K.TFAPEEISAMVLTK.M + Oxidation (M)		
	783.6500	1565.2854	2	23			R.ITPSYVAFTPEGER.L		
	830.1700	1658.3254	2	98			R.IINEPTAAAIAYGLDK.R		
	839.1600	1676.3054	2	73			K.NQLTSNPENTVFDAK.R		
	918.6300	1835.2454	2	75			K.SQIFSTASDNQPTVTIK.V		
1013	408.1300	814.2454	2	23	27	20	K.ESPLLFK.F + Glu->pyro-Glu (N-term E)	23	314
	417.1100	832.2054	2	(18)			K.ESPLLFK.F		
	444.1400	886.2654	2	21			K.EALLQASR.D		
	447.6700	893.3254	2	26			R.LFFLQVK.E		
	480.1800	958.3454	2	14			K.FVIKPIDK.K		
	481.1200	960.2254	2	45			R.KESPLLFK.F		
	482.1500	962.2854	2	50			R.ISQLEMAR.Q + Oxidation (M)		
	494.0700	986.1254	2	(51)			R.ALELEQER.K		
	494.0800	986.1454	2	51			R.ALELEQER.K		
	494.1000	986.1854	2	(32)			R.ALELEQER.K		
	494.1300	986.2454	2	(51)			R.ALELEQER.K		
	523.0300	1044.0454	2	(57)			K.ALTSELANAR.D		
	523.1400	1044.2654	2	75			K.ALTSELANAR.D		
	523.1600	1044.3054	2	(55)			K.ALTSELANAR.D		
	531.0500	1060.0854	2	31			R.EKEELMER.L + Glu->pyro-Glu (N-term E); Oxidation (M)		
	540.1100	1078.2054	2	29			R.EDAVLEYLK.I		
	591.6400	1181.2654	2	68			K.APDFVFYAPR.L		
	595.5900	1189.1654	2	(15)			K.AQMVQEDLEK.T		
	603.6000	1205.1854	2	61			K.AQMVQEDLEK.T + Oxidation (M)		
	616.1300	1230.2454	2	66			K.AQQELEEQTR.R		
	666.5700	1331.1254	2	37			R.KPDTIEVQQMK.A + Oxidation (M)		

	680.1600	1358.3054	2	55			K.KAQQELEEQTR.R		
	744.6600	1487.3054	2	36			R.RKPDTIEVQQMK.A + Oxidation (M)		
	496.8400	1487.4982	2	(12)			R.RKPDTIEVQQMK.A + Oxidation (M)		
	860.1700	1718.3254	2	92			K.TQEQLALEMAELTAR.I + Oxidation (M)		
	924.0900	1846.1654	2	67			K.KTQEQLALEMAELTAR.I + Oxidation (M)		
	616.4900	1846.4482	3	(10)			K.KTQEQLALEMAELTAR.I + Oxidation (M)		
955	480.1800	958.3454	2	14	6	5	K.FVIKPIDK.K	30	288
	591.6400	1181.2654	2	68			K.APDFVFYAPR.L		
	666.5700	1331.1254	2	37			R.KPDTIEVQQMK.A + Oxidation (M)		
	744.6600	1487.3054	2	36			R.RKPDTIEVQQMK.A + Oxidation (M)		
	496.8400	1487.4982	3	(12)			R.RKPDTIEVQQMK.A + Oxidation (M)		
	793.5300	2377.5682	3	6			-.GMVAQDLEMYGINYFEIKNK.K + Oxidation (M)		
955	444.1700	886.3254	2	25	13	10	K.EALLQASR.D	15	277
	481.1200	960.2254	2	10			R.KESPLLFK.F		
	482.1400	962.2654	2	50			R.ISQLEMAR.Q + Oxidation (M)		
	494.1100	986.2054	2	50			R.ALELEQER.K		
	494.1500	986.2854	2	(45)			R.ALELEQER.K		
	523.0900	1044.1654	2	(9)			K.ALTSELANAR.D		
	523.1700	1044.3254	2	54			K.ALTSELANAR.D		
	603.6100	1205.2054	2	46			K.AQMVQEDLEK.T + Oxidation (M)		
	616.0900	1230.1654	2	61			K.AQQELEEQTR.R		
	496.8300	1487.4682	3	29			R.RKPDTIEVQQMK.A + Oxidation (M)		
	860.1600	1718.3054	2	60			K.TQEQLALEMAELTAR.I + Oxidation (M)		
	573.8300	1718.4682	3	(49)			K.TQEQLALEMAELTAR.I + Oxidation (M)		
	616.5000	1846.4782	3	46			K.KTQEQLALEMAELTAR.I + Oxidation (M)		
832	443.6800	885.3454	2	(23)	19	16	R.RLSELLR.Y	25	343
	443.6800	885.3454	2	23			R.RLSELLR.Y		
	540.5600	1079.1054	2	23			R.APFDLFENK.K		
	576.5700	1151.1254	2	47			K.YIDQEELNK.T		
	597.6600	1193.3054	2	33			K.IDIIPNPQER.T		
	618.5500	1235.0854	2	35			R.RAPFDLFENK.K		
	621.6200	1241.2254	2	37			K.ADLINNLGTIAK.S		
	625.1200	1248.2254	2	34			K.EQVANSAFVER.V		
	638.0900	1274.1654	2	(60)			R.ELISNASDALDK.I		
	638.1000	1274.1854	2	(19)			R.ELISNASDALDK.I		
	638.1300	1274.2454	2	60			R.ELISNASDALDK.I		
	656.0400	1310.0654	2	45			K.EDQTEYLEER.R		
	683.1500	1364.2854	2	71			R.TLTLVDTGIGMTK.A + Oxidation (M)		
	708.6000	1415.1854	2	18			K.EGLELPEDEEEK.K		
	747.5400	1493.0654	2	71			K.IEDVGSDEEDDSGK.D		
	757.1200	1512.2254	2	70			R.GVVDSEDLPLNISR.E		
	515.4000	1543.1782	3	11			R.ELISNASDALDKIR.Y		
	924.1500	1846.2854	2	76			R.NPDDITQEEYGEFYK.S		
	1178.4000	3532.1782	3	74			K.LGLGIDEDEVAAEEPNAAVPDEIPPLEGDEDASR.M		
1162	451.6200	901.2254	2	12	12	8	R.STLEPVEK.A	16	344
	555.5800	1109.1454	2	(51)			K.LLQDFFDGR.D		
	555.6000	1109.1854	2	(44)			K.LLQDFFDGR.D		
	555.6300	1109.2454	2	55			K.LLQDFFDGR.D		
	599.1300	1196.2454	2	23			K.DAGVIAGLNVLR.I		
	614.5700	1227.1254	2	56			K.VEIIANDQGNR.T		
	615.0900	1228.1654	2	(6)			K.VEIIANDQGNR.T		
	619.5400	1237.0654	2	52			K.FGDPVVQSDMK.H + Oxidation (M)		
	744.0000	1485.9854	2	90			R.TTPSYVAFTDTER.L		
	829.6800	1657.3454	2	68			K.NQVALNPQNTVFDAK.R		
	830.1000	1658.1854	2	(47)			K.NQVALNPQNTVFDAK.R		
	844.5300	1687.0454	2	80			R.IINEPTAAAIAYGLDR.T		
1162	411.1500	820.2854	2	35	8	7	R.TFLVGER.V	17	342
	543.1100	1084.2054	2	27			K.STFVLDEFK.R		
	559.6700	1117.3254	2	47			R.ILGLLDAYLK.T		
	674.0500	1346.0854	2	78			K.ALIAAQYSGAQVR.V		
	722.5900	1443.1654	2	69			K.LDPGSEETQTLVR.E		
	524.7700	1571.2882	3	43			R.KLDPGSEETQTLVR.E		
	786.4000	2356.1782	3	(8)			K.AAAPAPEEEMDECEQALAAEPK.A		
	792.0400	2373.0982	3	18			K.AAAPAPEEEMDECEQALAAEPK.A + Oxidation (M)		
705	438.6500	875.2854	2	34	33	23	K.TFEINPR.H	30	186
	438.6900	875.3654	2	(22)			K.TFEINPR.H		
	460.1800	918.3454	2	16			K.NKEIFLR.E		
	482.2300	962.4454	2	39			K.LIINSLYK.N		
	491.6300	981.2454	2	34			K.SGTSEFLNK.M		
	497.1700	992.3254	2	(42)			R.SGYLLPDTK.A		
	497.1900	992.3654	2	51			R.SGYLLPDTK.A		
	508.1600	1014.3054	2	30			R.GLFDEYGSK.K		
	524.1200	1046.2254	2	40			K.IYFMAGSSR.K + Oxidation (M)		
	524.1500	1046.2854	2	(36)			K.IYFMAGSSR.K + Oxidation (M)		
	541.1900	1080.3654	2	52			K.FAFQAEVNR.M		
	543.2700	1084.5254	2	52			R.LSLNIDPDAK.V		
	575.6200	1149.2254	2	(20)			K.EAESSPFVER.L		
	575.6400	1149.2654	2	34			K.EAESSPFVER.L		
	584.1900	1166.3654	2	24			K.EGVKFDESEK.T		
	594.2600	1186.5054	2	(43)			K.SILFVPTSAPR.G		
	594.2700	1186.5254	2	43			K.SILFVPTSAPR.G		
	638.2300	1274.4454	2	60			R.ELISNASDALDK.I		
	639.6800	1277.3454	2	34			R.KEAESSPFVER.L		
	426.8500	1277.5282	3	(26)			R.KEAESSPFVER.L		
	645.1000	1288.1854	2	18			K.DISTNYYASQK.K		
	743.2900	1484.5654	2	(67)			K.GVVDSDDLPLNVSR.E		
	743.3700	1484.7254	2	76			K.GVVDSDDLPLNVSR.E		
	763.2300	1524.4454	2	79			K.EEASDYLELDTIK.N		
	515.5400	1543.5982	3	34			R.ELISNASDALDKIR.L		
	867.6900	1733.3654	2	(43)			-.DDEVDVDGTVEEDLGK.S		
	867.7100	1733.4054	2	85			-.DDEVDVDGTVEEDLGK.S		
	869.2400	1736.4654	2	17			K.TETVEEPMEEEEAAK.E + Oxidation (M)		
	893.2800	1784.5454	2	72			R.EEEAIQLDGLNASQIR.E		
	1023.3500	2044.6854	2	83			R.LISLTDENALSGNEELTVK.I		
	1023.8800	2045.7454	2	(43)			R.LISLTDENALSGNEELTVK.I		
	909.9700	2726.8882	3	83			R.TDDEVVQREEEAIQLDGLNASQIR.E		
	910.2800	2727.8182	3	(66)			R.TDDEVVQREEEAIQLDGLNASQIR.E		
1278	401.1400	800.2654	2	27	20	15	K.TIADIIR.T	28	303
	426.1100	850.2054	2	33			K.SMIEISR.T + Oxidation (M)		
	487.5800	973.1454	2	(29)			K.EILSEVER.N		
	487.6300	973.2454	2	31			K.EILSEVER.N		
	561.6500	1121.2854	2	(44)			K.ALDDMISTLK.K + Oxidation (M)		
	561.6600	1121.3054	2	46			K.ALDDMISTLK.K + Oxidation (M)		
	563.0200	1124.0254	2	14			K.MVQFEENGR.K + Oxidation (M)		
	583.6500	1165.2854	2	(31)			R.AVAQALEVIPR.T		
	583.7200	1165.4254	2	62			R.AVAQALEVIPR.T		
	593.2200	1184.4254	2	68			K.TAVETAVLLLR.I		
	624.6000	1247.1854	2	57			K.GESQTDIEITR.E		
	624.6200	1247.2254	2	(48)			K.GESQTDIEITR.E		
	625.6800	1249.3454	2	35			R.KALDDMISTLK.K + Oxidation (M)		
	640.2000	1278.3854	2	51			R.IVLLDSSLEYK.K		

	459.7800	1376.3182	3	46			K.KGESQTDIEITR.E		
	702.0400	1402.0654	2	18			K.GISDLAQHYLMR.A		
	708.6800	1415.3454	2	56			R.EDDVGTGAGLLEIK.K		
	726.8000	2177.3782	3	62			K.MLLDPMGGIVMTNDGNAILR.E + 3 Oxidation (M)		
	1090.1400	2178.2654	2	(8)			K.MLLDPMGGIVMTNDGNAILR.E + 3 Oxidation (M)		
	880.2100	2637.6082	3	43			K.ISIPVDISDSDMMLNIINSSITTK.A + 2 Oxidation (M)		
1469	422.5900	843.1654	2	20	18	12	R.LVGIISSR.D	21	346
	482.1000	962.1854	2	39			R.VRDVFEAK.A		
	486.5500	971.0854	2	28			K.EANEILQR.S		
	486.6000	971.1854	2	(28)			K.EANEILQR.S		
	486.6300	971.2454	2	(27)			K.EANEILQR.S		
	531.0500	1060.0854	2	19			R.AMMYSGELK.F + 2 Oxidation (M)		
	550.8200	1099.6254	2	23			R.YFSEADKIK.V		
	578.6100	1155.2054	2	(60)			K.NLIDAGVDALR.V		
	578.6100	1155.2054	2	70			K.NLIDAGVDALR.V		
	579.6500	1157.2854	2	60			K.VAQGVSGAVQDK.G		
	663.1300	1324.2454	2	23			R.EDLVVAPAGITLK.E		
	741.1900	1480.3654	2	106			K.REDLVVAPAGITLK.E		
	741.2000	1480.3854	2	(80)			K.REDLVVAPAGITLK.E		
	494.4900	1480.4482	3	(48)			K.REDLVVAPAGITLK.E		
	846.6300	1691.2454	2	61			K.YEQGFITDPVVLSPK.D		
	890.1900	1778.3654	2	87			K.LPIVNEDDELVAIIAR.T		
	910.5900	1819.1654	2	46			K.KYEQGFITDPVVLSPK.D		
	607.4400	1819.2982	3	(36)			K.KYEQGFITDPVVLSPK.D		
983	452.1600	902.3054	2	35	20	17	K.AESLQVTR.G	16	235
	487.2300	972.4454	2	33			K.LLIIGTTSR.K		
	500.1400	998.2654	2	43			K.MIGFSETAK.C + Oxidation (M)		
	524.1100	1046.2054	2	42			K.MEIGLPDEK.G + Oxidation (M)		
	542.0300	1082.0454	2	18			K.VVNGPEILNK.Y		
	551.2800	1100.5454	2	36			R.KLLIIGTTSR.K		
	569.1200	1136.2254	2	55			K.YVGESEANIR.K		
	571.7100	1141.4054	2	32			R.LLDYVPIGPR.F		
	573.1200	1144.2254	2	(54)			K.AENSSLNLIGK.A		
	573.2000	1144.3854	2	60			K.AENSSLNLIGK.A		
	604.1200	1206.2254	2	56			K.LFADAEEEQR.R		
	624.1800	1246.3454	2	42			K.DIESMDPSILK.G		
	420.8100	1259.4082	3	(30)			K.MEIGLPDEKGR.L + Oxidation (M)		
	420.8700	1259.5882	3	42			K.MEIGLPDEKGR.L + Oxidation (M)		
	632.1700	1262.3254	2	(38)			K.DIESMDPSILK.G + Oxidation (M)		
	668.1500	1334.2854	2	58			R.KLFADAEEEQR.R		
	455.1400	1362.3982	3	18			K.LFADAEEEQRR.L		
	486.5200	1456.5382	3	(39)			R.VLDDGELLVQQTK.N		
	729.2900	1456.5654	2	69			R.VLDDGELLVQQTK.N		
	583.7600	1748.2582	3	56			K.VEVDMEKAESLQVTR.G + Oxidation (M)		
781	411.1100	820.2054	2	45	27	25	K.FGEEIAR.L	26	223
	450.0600	898.1054	2	(17)			R.EGLENDLK.S + Glu->pyro-Glu (N-term E)		
	450.1500	898.2854	2	22			K.FTDLFEK.M		
	459.1200	916.2254	2	27			R.EGLENDLK.S		
	465.6900	929.3654	2	36			K.DLQQSIAR.E		
	478.2000	954.3854	2	43			K.EVFPVLAAK.H + Glu->pyro-Glu (N-term E)		
	487.1600	972.3054	2	(31)			K.EVFPVLAAK.H		
	487.7200	973.4254	2	45			R.EILDESLR.L		
	488.2300	974.4454	2	45			K.ETVLSALSR.E		
	499.1800	996.3454	2	25			K.ELPELLQR.N		
	499.1800	996.3454	2	(24)			K.ELPELLQR.N		
	515.1300	1028.2454	2	39			R.YDEYVNVK.D		
	522.6600	1043.3054	2	34			K.SVNFDMTSK.F + Oxidation (M)		
	532.7200	1063.4254	2	52			K.LALASLGYEK.S		
	559.1400	1116.2654	2	49			K.SLLSNLDEVK.K		
	585.2400	1168.4654	2	31			K.STPVNVPISQK.F		
	623.2400	1244.4654	2	43			K.SLLSNLDEVKK.E		
	650.2900	1298.5654	2	19			K.TSEVDLAKPLVK.F		
	659.0600	1316.1054	2	9			R.TPSNELYKPLR.A		
	660.7200	1319.4254	2	75			K.TMQGSEVVNVLK.S + Oxidation (M)		
	698.7200	1395.4254	2	73			K.FYNELTEILVR.F		
	766.7400	1531.4654	2	72			R.LLDEEEATDNDLR.A		
	548.4700	1642.3882	3	41			K.QSNNEANLREEVLK.N		
	864.3100	1726.6054	2	69			R.SVIEQGGIQTVDQLIK.E		
	576.5800	1726.7182	3	(34)			R.SVIEQGGIQTVDQLIK.E		
	577.7800	1730.3182	3	17			R.LLDEEEATDNDLRAK.F		
	792.3300	2373.9682	2	14			R.TMPPTKPQPPARPPPPVLPANR.A + Oxidation (M)		
1289	405.1300	808.2454	2	24	23	19	K.DFDTALK.H	27	294
	414.6000	827.1854	2	40			R.IGNSYFK.E		
	441.6500	881.2854	2	27			R.ELIEQLR.N + Glu->pyro-Glu (N-term E)		
	502.7000	1003.3854	2	57			K.AAALEFLNR.F		
	504.6300	1007.2454	2	36			K.ALDLDSSCK.E		
	509.6100	1017.2054	2	38			R.LILEQMQK.D + Oxidation (M)		
	533.1600	1064.3054	2	23			R.TLLSDPTYR.E		
	558.7100	1115.4054	2	65			K.LMDVGLIAIR.- + Oxidation (M)		
	566.7300	1131.4454	2	50			R.KAAALEFLNR.F		
	607.6100	1213.2054	2	(21)			R.IGNSYFKEEK.Y		
	405.4500	1213.3282	3	29			R.IGNSYFKEEK.Y		
	443.1500	1326.4282	3	(18)			K.AAALEAMKDYTK.A + Oxidation (M)		
	664.6100	1327.2054	2	34			K.AAALEAMKDYTK.A + Oxidation (M)		
	744.7700	1487.5254	2	54			R.LAYINPDLALEEK.N		
	838.2000	1674.3854	2	44			K.FMNPFNMPNLYQK.L + 2 Oxidation (M)		
	601.1100	1800.3082	3	16			K.ETKPEPMEEDLPENK.K + Oxidation (M)		
	601.8000	1802.3782	3	42			R.KFMNPFNMPNLYQK.L + 2 Oxidation (M)		
	637.6200	1909.8382	3	6			-.MESGSPMGEVEISRTIR.T + 2 Oxidation (M)		
	643.7800	1928.3182	3	31			K.ETKPEPMEEDLPENKK.Q + Oxidation (M)		
	969.1500	1936.2854	2	61			R.AMADPEVQQIMSDPAMR.L + 3 Oxidation (M)		
	969.1600	1936.3054	2	(48)			R.AMADPEVQQIMSDPAMR.L + 3 Oxidation (M)		
	646.4700	1936.3882	3	(55)			R.AMADPEVQQIMSDPAMR.L + 3 Oxidation (M)		
	698.4800	2092.4182	3	60			R.RAMADPEVQQIMSDPAMR.L + 3 Oxidation (M)		
1236	451.1300	900.2454	2	44	8	8	K.LEAALGEAK.K	16	267
	513.2300	1024.4454	2	10			K.EGDLIAAQAR.L + Glu->pyro-Glu (N-term E)		
	514.6800	1027.3454	2	46			R.LADALQELR.A		
	583.1300	1164.2454	2	71			K.AAYEAELGDAR.K		
	680.2200	1358.4254	2	69			R.SGAQASSTPLSPTR.I		
	716.2400	1430.4654	2	44			R.IDSLSAQLSQLQK.Q		
	754.2000	1506.3854	2	71			R.TALINSTGEEVAMR.K + Oxidation (M)		
	776.6500	1551.2854	2	12			K.SNEDQSMGNWQIK.R + Oxidation (M)		
1236	509.6300	1017.2454	2	38	6	5	R.LILEQMQK.D + Oxidation (M)		
	558.7500	1115.4854	2	51			K.LMDVGLIAIR.- + Oxidation (M)		
	744.7200	1487.4254	2	42			R.LAYINPDLALEEK.N		
	543.0200	1626.0382	2	9			R.VAEVRSSLHSWSLR.W		
	969.2200	1936.4254	2	(47)			R.AMADPEVQQIMSDPAMR.L + 3 Oxidation (M)		
	646.4900	1936.4482	2	66			R.AMADPEVQQIMSDPAMR.L + 3 Oxidation (M)		
1387	498.5800	995.1454	2	42	12	9	K.ASSELFSQK.T	15	254
	498.5900	995.1654	2	(29)			K.ASSELFSQK.T		
	529.6600	1057.3054	2	44			K.LEDTYFDR.D		

	609.2000	1216.3854	2	58			R.LEVGTETIIDK.S		
	651.6700	1301.3254	2	30			K.VTQDATPGSALDK.I		
	697.7200	1393.4254	2	46			R.TGVAPDVFAENMK.L + Oxidation (M)		
	750.2300	1498.4454	2	91			K.NSIYSGLEAFGDVK.L		
	503.4700	1507.3882	3	(34)			R.MLLNDFLNDQNR.D + Oxidation (M)		
	754.7400	1507.4654	2	70			R.MLLNDFLNDQNR.D + Oxidation (M)		
	755.2100	1508.4054	2	(22)			R.MLLNDFLNDQNR.D + Oxidation (M)		
	510.5000	1528.4782	3	22			K.LEDTYFDRDVEK.A		
	584.5000	1750.4782	3	61			R.MLLNDFLNDQNRDK.N + Oxidation (M)		
1464	410.5600	819.1054	2	(16)	26	13	R.DLQSSDR.L	26	258
	410.5600	819.1054	2	(17)			R.DLQSSDR.L		
	410.5900	819.1654	2	(16)			R.DLQSSDR.L		
	410.6300	819.2454	2	20			R.DLQSSDR.L		
	412.1200	822.2254	2	15			R.EDQIYR.I		
	432.0800	862.1454	2	(32)			K.LPDAYER.L		
	432.0900	862.1654	2	(32)			K.LPDAYER.L		
	432.0900	862.1654	2	32			K.LPDAYER.L		
	432.1400	862.2654	2	(32)			K.LPDAYER.L		
	432.1500	862.2854	2	(32)			K.LPDAYER.L		
	441.6600	881.3054	2	15			K.QSEPFFK.A		
	529.6800	1057.3454	2	26			R.IIVEKPFGR.D		
	553.6400	1105.2654	2	(40)			R.GPTEADELMK.R + Oxidation (M)		
	553.6400	1105.2654	2	57			R.GPTEADELMK.R + Oxidation (M)		
	553.6400	1105.2654	2	(56)			R.GPTEADELMK.R + Oxidation (M)		
	553.6700	1105.3254	2	(40)			R.GPTEADELMK.R + Oxidation (M)		
	566.5700	1131.1254	2	29			K.GYLDDPTVPR.G		
	574.6800	1147.3454	2	26			R.VQPNEAVYTK.M		
	632.5600	1263.1054	2	(26)			K.EMVQNLMVLR.F + 2 Oxidation (M)		
	632.6100	1263.2054	2	29			K.EMVQNLMVLR.F + 2 Oxidation (M)		
	637.1500	1272.2854	2	(43)			R.GGYFDEFGIIR.D		
	637.1500	1272.2854	2	54			R.GGYFDEFGIIR.D		
	832.7700	1663.5254	2	48			R.DGLLPENTFIVGYAR.S		
	912.8000	1823.5854	2	28			R.LFYLALPPTVYEAVTK.N		
	608.8800	1823.6182	3	(14)			R.LFYLALPPTVYEAVTK.N		
	964.2200	1926.4254	2	46			R.GSTTATFAAVVLYVENER.W		
675	443.1900	884.3654	2	23	9	7	K.QILGVIDK.K	11	296
	615.5600	1229.1054	2	69			R.LNQDQLDAVSK.Y		
	615.6200	1229.2254	2	(64)			R.LNQDQLDAVSK.Y		
	642.1600	1282.3054	2	58			R.SFMALSQDIQK.T + Oxidation (M)		
	660.8200	1319.6254	2	57			K.TVLELQYVLDK.L		
	449.4700	1345.3882	3	23			K.LVDPERDMSLR.L + Oxidation (M)		
	728.1000	1454.1854	2	61			K.YQEVTNNLEFAK.E		
	728.1600	1454.3054	2	(61)			K.YQEVTNNLEFAK.E		
	760.0400	1518.0654	2	75			R.QFMAETQFTSGEK.E + Oxidation (M)		
675	406.1800	810.3454	2	42	17	13	K.VQQLVPK.R	17	203
	432.6100	863.2054	2	48			K.ALDFIASK.G		
	546.6400	1091.2654	2	26			K.NFITAEELR.R		
	580.1100	1158.2054	2	19			K.STLPDADRER.E		
	685.0000	1367.9854	2	43			K.GISQEQMQEFR.A + Oxidation (M)		
	715.1900	1428.3654	2	63			R.TINEVENQILTR.D		
	725.0600	1448.1054	2	(17)			R.LSNRPAFMPSEGK.M + Oxidation (M)		
	483.8100	1448.4082	2	32			R.LSNRPAFMPSEGK.M + Oxidation (M)		
	757.6800	1513.3454	2	69			K.LVSIGAEEIVDGNAK.M		
	505.4800	1513.4182	3	(9)			K.LVSIGAEEIVDGNAK.M		
	769.1200	1536.2254	2	64			R.FAIQDISVEETSAK.E		
	559.1400	1674.3982	3	48			K.VLAGDKNFITAEELR.R		
	871.1300	1740.2454	2	63			R.ETTDTDTADQVIASFK.V		
	581.1300	1740.3682	3	(44)			R.ETTDTDTADQVIASFK.V		
	581.1300	1740.3682	3	(35)			R.ETTDTDTADQVIASFK.V		
	635.0500	1902.1282	3	18			R.KDDPVTNLNNAFEVAEK.Y		
	641.0800	1920.2182	3	20			K.AGTQIENIDEDFRDGLK.L		
803	472.6700	943.3254	2	(16)	28	21	R.ESIESEIR.R + Glu->pyro-Glu (N-term E)	26	234
	474.2100	946.4054	2	46			R.KGDIFLVR.G		
	481.6200	961.2254	2	28			R.ESIESEIR.R		
	481.6500	961.2854	2	(28)			R.ESIESEIR.R		
	501.6900	1001.3654	2	17			K.EMVELPLR.H + Oxidation (M)		
	516.6600	1031.3054	2	48			K.GDDLSTAILK.Q		
	525.1600	1048.3054	2	48			K.DVDLEFLAK.M		
	534.1900	1066.3654	2	45			K.MDELQLFR.G + Oxidation (M)		
	538.1700	1074.3254	2	77			K.LAGESESNLR.K		
	559.6200	1117.2254	2	18			R.ESIESEIRR.E		
	586.7100	1171.4054	2	31			R.GILLYGPPGTGK.T		
	621.7200	1241.4254	2	21			R.EVDIGIPDATGR.L		
	723.8000	1445.5854	2	76			R.IVSQLLTLMDGLK.Q + Oxidation (M)		
	759.1600	1516.3054	2	57			K.YEMFAQTLQQSR.G + Oxidation (M)		
	778.7600	1555.5054	2	53			R.LDQLIYIPLPDEK.S		
	823.2100	1644.4054	2	70			R.KYEMFAQTLQQSR.G + Oxidation (M)		
	549.1800	1644.5182	3	(44)			R.KYEMFAQTLQQSR.G + Oxidation (M)		
	856.2500	1710.4854	2	(56)			R.VINQILTEMDGMSTK.K + 2 Oxidation (M)		
	571.2300	1710.6682	3	(34)			R.VINQILTEMDGMSTK.K + 2 Oxidation (M)		
	856.7400	1711.4654	2	64			R.VINQILTEMDGMSTK.K + 2 Oxidation (M)		
	600.8300	1799.4682	3	22			R.LDQLIYIPLPDEKSR.V		
	905.8000	1809.5854	2	69			K.NAPAIIFIDELDAIAPK.R		
	604.5600	1810.6582	3	(22)			K.NAPAIIFIDELDAIAPK.R		
	962.1300	1922.2454	2	19			R.QAAPCVLFFDELDSIAK.A		
	1085.3000	2168.5854	2	88			R.LIVDEAINEDNSVVSLSQPK.M		
	723.9500	2168.8282	3	(36)			R.LIVDEAINEDNSVVSLSQPK.M		
	1086.1900	2170.3654	2	42			R.QTNPSAMEVEEDDPVPEIR.R + Oxidation (M)		
	724.8600	2171.5582	3	(37)			R.QTNPSAMEVEEDDPVPEIR.R + Oxidation (M)		
620	559.1700	1116.3254	2	53	11	10	K.LKETAESVLK.K	14	297
	567.1400	1132.2654	2	20			K.QSLTMDPVVK.S + Oxidation (M)		
	620.1600	1238.3054	2	46			R.AFSDPFVEAEK.S		
	661.2500	1320.4854	2	87			K.VLATAFDTTLGGR.K		
	699.6700	1397.3254	2	27			K.FLEMCNDLLAR.V + Oxidation (M)		
	734.6500	1467.2854	2	34			R.EMLNLYIENEGK.M + Oxidation (M)		
	748.6500	1495.2854	2	51			R.AGGIETIANEYSDR.C		
	760.3100	1518.6054	2	66			K.ELSTTLNADEAVTR.G		
	760.7500	1519.4854	2	(65)			K.ELSTTLNADEAVTR.G		
	799.7700	1597.5254	2	108			R.NFTTEQVTAMLLSK.L + Oxidation (M)		
	586.6800	1757.0182	3	11			R.LMNETTAVALAYGIYK.Q		
620	540.1900	1078.3654	2	32	6	5	R.AFNDPFIQK.E	6	163
	560.2300	1118.4454	2	47			R.FVVQNVSAQK.D		
	560.2800	1118.5454	2	(42)			R.FVVQNVSAQK.D		
	661.2600	1320.5054	2	24			K.VLGTAFDPFLGGK.N		
	738.2200	1474.4254	2	72			K.DISTTLNADEAVAR.G		
	751.2400	1500.4654	2	57			K.VEDVSAVEIVGGATR.I		
1130	402.6400	803.2654	2	24	32	23	K.ITITNDK.G	37	264
	472.7100	943.4054	2	29			K.VCNPIITK.L		
	495.1500	988.2854	2	41			R.LSKEDIER.M		
	497.1700	992.3254	2	45			K.EIAEAYLGK.T		
	541.1500	1080.2854	2	28			K.LLQDFFNGK.E		

	600.2100	1198.4054	2	42			K.DAGTIAGLNVLR.I		
	614.6700	1227.3254	2	57			K.VEIIANDQGNR.T		
	615.0900	1228.1654	2	(19)			K.VEIIANDQGNR.T		
	627.1700	1252.3254	2	48			R.FEELNADLFR.G		
	635.6100	1269.2054	2	61			R.FDDAVVQSDMK.H + Oxidation (M)		
	660.1400	1318.2654	2	72			K.NSLESYAFNMK.A + Oxidation (M)		
	660.1400	1318.2654	2	(60)			K.NSLESYAFNMK.A + Oxidation (M)		
	660.6100	1319.2054	2	(44)			K.NSLESYAFNMK.A + Oxidation (M)		
	476.3800	1426.1182	3	36			R.RFDDAVVQSDMK.H + Oxidation (M)		
	482.1200	1443.3382	3	12			K.ELEKVCNPIITK.L		
	494.1800	1479.5182	3	41			R.ARFEELNADLFR.G		
	744.2100	1486.4054	2	77			R.TTPSYVAFTDTER.L		
	522.7900	1565.3482	3	44			K.LLQDFFNGKELNK.S		
	816.6800	1631.3454	2	61			K.SFYPEEVSSMVLTK.M + Oxidation (M)		
	830.2700	1658.5254	2	80			R.IINEPTAAAIAYGLDK.K		
	554.1200	1659.3382	3	(50)			R.IINEPTAAAIAYGLDK.K		
	555.7500	1664.2282	3	(35)			K.NQVAMNPTNTVFDAK.R + Oxidation (M)		
	833.3700	1664.7254	2	71			K.NQVAMNPTNTVFDAK.R + Oxidation (M)		
	833.7000	1665.3854	2	(40)			K.NQVAMNPTNTVFDAK.R + Oxidation (M)		
	894.2200	1786.4254	2	57			R.IINEPTAAAIAYGLDKK.V		
	596.5200	1786.5382	3	(27)			R.IINEPTAAAIAYGLDKK.V		
	991.2600	1980.5054	2	(36)			K.TVTNAVVTVPAYFNDSQR.Q		
	661.1900	1980.5482	3	41			K.TVTNAVVTVPAYFNDSQR.Q		
	1130.3200	2258.6254	2	77			K.SINPDEAVAYGAAVQAAILSGDK.S		
	753.9300	2258.7682	3	(50)			K.SINPDEAVAYGAAVQAAILSGDK.S		
	1130.7900	2259.5654	2	(44)			K.SINPDEAVAYGAAVQAAILSGDK.S		
	1126.8600	3377.5582	3	17			K.LYQSAGGMPGGMPGGFPGGGAPPSGGASSGPTIEEVD.- + 2 Oxidation (M)		
670	450.6300	899.2454	2	43	19	10	R.LVSVLQNK.M	13	272
	450.7000	899.3854	2	(29)			R.LVSVLQNK.M		
	450.7000	899.3854	2	(27)			R.LVSVLQNK.M		
	704.6300	1407.2454	2	(40)			R.AVFDETYPDPVR.V		
	704.6700	1407.3254	2	54			R.AVFDETYPDPVR.V		
	717.5600	1433.1054	2	100			K.AEEIANEMIEAAK.A + Oxidation (M)		
	717.6400	1433.2654	2	(59)			K.AEEIANEMIEAAK.A + Oxidation (M)		
	479.0400	1434.0982	3	(6)			K.AEEIANEMIEAAK.A + Oxidation (M)		
	738.7500	1475.4854	2	83			K.DIINEEEVQFLK.T		
	749.6200	1497.2254	2	82			K.VGAEDADGIDMAYR.V + Oxidation (M)		
	749.6300	1497.2454	2	(78)			K.VGAEDADGIDMAYR.V + Oxidation (M)		
	756.0900	1510.1654	2	77			K.GLVVDMDGFEEER.K + Oxidation (M)		
	756.1500	1510.2854	2	(64)			K.GLVVDMDGFEEER.K + Oxidation (M)		
	521.4800	1561.4182	3	57			K.KAEEIANEMIEAAK.A + Oxidation (M)		
	521.7700	1562.2882	3	(25)			K.KAEEIANEMIEAAK.A + Oxidation (M)		
	571.8700	1712.5882	3	71			R.TITVALADGGRPDNTGR.G		
	572.1500	1713.4282	3	(70)			R.TITVALADGGRPDNTGR.G		
	938.7400	1875.4654	2	29			K.MALELLTQEFGIPIER.L + Oxidation (M)		
	835.2200	2502.6382	3	64			K.QFIDSNPNQPLVILEMESGASAK.A + Oxidation (M)		
594	407.1500	812.2854	2	32	25	17	K.NIILGGVK.A	19%	213
	492.6100	983.2054	2	28			R.DEFEGLFK.Q		
	625.6100	1249.2054	2	(37)			R.AENYDIPSADR.H		
	625.6300	1249.2454	2	39			R.AENYDIPSADR.H		
	630.2700	1258.5254	2	56			R.LQTSSVLVSGLR.G		
	630.2700	1258.5254	2	(49)			R.LQTSSVLVSGLR.G		
	652.6000	1303.1854	2	54			R.LDQPMTEIVSR.V + Oxidation (M)		
	652.6500	1303.2854	2	(46)			R.LDQPMTEIVSR.V + Oxidation (M)		
	457.5300	1369.5682	3	23			K.ATLPSPDKLPGFK.M		
	723.1700	1444.3254	2	32			K.DNPGVVTCLDEAR.H		
	782.7100	1563.4054	2	5			-.MRSLPQMSSSPLSK.K + Oxidation (M)		
	530.5200	1588.5382	3	(20)			K.ERLDQPMTEIVSR.V + Oxidation (M)		
	530.6200	1588.8382	3	21			K.ERLDQPMTEIVSR.V + Oxidation (M)		
	808.7400	1615.4654	2	40			K.QPAENVNQYLTDPK.F		
	541.9200	1622.7382	3	(61)			R.LAGTQPLEVLEAVQR.S		
	812.3900	1622.7654	2	69			R.LAGTQPLEVLEAVQR.S		
	828.2100	1654.4054	2	74			R.YDGQVAVFGSDLQEK.L		
	828.2100	1654.4054	2	(69)			R.YDGQVAVFGSDLQEK.L		
	904.7700	1807.5254	2	61			R.ALPAVQQNNLDEDLIR.K		
	942.3100	1882.6054	2	(67)			R.NEEDAAELVALAQAVNAR.A		
	942.3100	1882.6054	2	81			R.NEEDAAELVALAQAVNAR.A		
	954.7900	1907.5654	2	47			K.SLVASLAEPDFVVTDFAK.F		
	644.9000	1931.6782	3	70			R.AAVATFLQSVQVPEFTPK.S		
	966.8500	1931.6854	2	(47)			R.AAVATFLQSVQVPEFTPK.S		
	646.2000	1935.5782	3	41			R.ALPAVQQNNLDEDLIRK.L		
594	408.7400	815.4654	2	21	15	10	R.FIDFFK.R	14	252
	450.6300	899.2454	2	46			R.LVSVLQNK.M		
	450.6700	899.3254	2	(34)			R.LVSVLQNK.M		
	450.6900	899.3654	2	(43)			R.LVSVLQNK.M		
	704.7000	1407.3854	2	57			R.AVFDETYPDPVR.V		
	717.6300	1433.2454	2	75			K.AEEIANEMIEAAK.A + Oxidation (M)		
	717.6400	1433.2654	2	(69)			K.AEEIANEMIEAAK.A + Oxidation (M)		
	738.7200	1475.4254	2	67			K.DIINEEEVQFLK.T		
	750.0800	1498.1454	2	72			K.VGAEDADGIDMAYR.V + Oxidation (M)		
	756.1400	1510.2654	2	77			K.GLVVDMDGFEEER.K + Oxidation (M)		
	571.8100	1712.4082	3	54			R.TITVALADGGRPDNTGR.G		
	857.2200	1712.4254	2	(12)			R.TITVALADGGRPDNTGR.G		
	938.2900	1874.5654	2	43			K.MALELLTQEFGIPIER.L + Oxidation (M)		
	626.2100	1875.6082	3	(30)			K.MALELLTQEFGIPIER.L + Oxidation (M)		
	911.7900	2732.3482	3	16			K.TKQFIDSNPNQPLVILEMESGASAK.A + Oxidation (M)		
626	438.6600	875.3054	2	28	29	16	K.ETAESVLK.K	25	214
	559.1700	1116.3254	2	(7)			K.LKETAESVLK.K		
	559.1700	1116.3254	2	28			K.LKETAESVLK.K		
	559.2400	1116.4654	2	(11)			K.LKETAESVLK.K		
	559.2900	1116.5654	2	(18)			K.LKETAESVLK.K		
	567.1300	1132.2454	2	(20)			K.QSLTMDPVVK.S + Oxidation (M)		
	567.1700	1132.3254	2	24			K.QSLTMDPVVK.S + Oxidation (M)		
	584.1900	1166.3654	2	27			K.MIMQDKLEK.E + 2 Oxidation (M)		
	620.1700	1238.3254	2	(21)			R.AFSDPFVEAEK.S		
	620.2000	1238.3854	2	44			R.AFSDPFVEAEK.S		
	432.4500	1294.3282	3	41			K.QDLPALEEKPR.N		
	648.2100	1294.4054	2	(6)			K.QDLPALEEKPR.N		
	648.2100	1294.4054	2	(31)			K.QDLPALEEKPR.N		
	432.5400	1294.5982	3	(38)			K.QDLPALEEKPR.N		
	661.1700	1320.3254	2	(76)			K.VLATAFDTTLGGR.K		
	661.2100	1320.4054	2	79			K.VLATAFDTTLGGR.K		
	709.6500	1417.2854	2	55			K.NAVEEYVYEMR.D + Oxidation (M)		
	709.7200	1417.4254	2	(53)			K.NAVEEYVYEMR.D + Oxidation (M)		
	734.7800	1467.5454	2	35			R.EMLNLYIENEGK.M + Oxidation (M)		
	746.7200	1491.4254	2	78			K.EDIYAVEIVGGATR.I		
	748.1600	1494.3054	2	(42)			R.AGGIETIANEYSDR.C		
	748.1800	1494.3454	2	59			R.AGGIETIANEYSDR.C		
	760.2100	1518.4054	2	55			K.ELSTTLNADEAVTR.G		
	799.7300	1597.4454	2	108			R.NFTTEQVTAMLLSK.L + Oxidation (M)		
	887.2300	1772.4454	2	105			R.LMNETTAVALAYGIYK.Q + Oxidation (M)		

	951.8400	1901.6654	2	(66)			K.SNLAYDIVQLPTGLTGIK.V		
	634.9100	1901.7082	3	71			K.SNLAYDIVQLPTGLTGIK.V		
	952.2900	1902.5654	2	(19)			K.SNLAYDIVQLPTGLTGIK.V		
	1122.8600	3365.5582	3	20			K.NAEQNGPVDGQGDNPGPQAAEQGTDTAVPSDSDK.K		
1053	459.6200	917.2254	2	29	37	27	K.VLEDSDLK.K	37	250
	493.5900	985.1654	2	(19)			R.LTPEEIER.M		
	493.6000	985.1854	2	(24)			R.LTPEEIER.M		
	493.6300	985.2454	2	25			R.LTPEEIER.M		
	523.7200	1045.4254	2	36			K.VLEDSDLKK.S		
	537.6800	1073.3454	2	32			K.ITITNDQNR.L		
	596.0900	1190.1654	2	33			K.VYEGERPLTK.D		
	605.6000	1209.1854	2	25			K.EFFNGKEPSR.G		
	614.6300	1227.2454	2	58			R.VEIIANDQGNR.I		
	617.1700	1232.3254	2	45			K.DAGTIAGLNVMR.I + Oxidation (M)		
	658.6900	1315.3654	2	49			R.NELESYAYSLK.N		
	665.1300	1328.2454	2	58			K.FEELNMDLFR.S + Oxidation (M)		
	690.6500	1379.2854	2	(41)			K.ELEEIVQPIISK.L + Glu->pyro-Glu (N-term E)		
	699.2900	1396.5654	2	47			K.ELEEIVQPIISK.L		
	730.7600	1459.5054	2	50			K.SDIDEIVLVGGSTR.I		
	510.1700	1527.4882	3	(24)			R.AKFEELNMDLFR.S + Oxidation (M)		
	764.7700	1527.5254	2	69			R.AKFEELNMDLFR.S + Oxidation (M)		
	514.3800	1540.1182	3	38			R.MVNDAEKFAEEDK.K + Oxidation (M)		
	776.7600	1551.5054	2	81			K.TFAPEEISAMVLTK.M + Oxidation (M)		
	783.7800	1565.5454	2	41			R.ITPSYVAFTPEGER.L		
	522.9100	1565.7082	3	(22)			R.ITPSYVAFTPEGER.L		
	530.1900	1587.5482	3	35			K.KSDIDEIVLVGGSTR.I		
	802.6500	1603.2854	2	18			K.TKPYIQVDIGGGQTK.T		
	830.2800	1658.5454	2	94			R.IINEPTAAAIAYGLDK.R		
	559.8100	1676.4082	3	(40)			K.NQLTSNPENTVFDAK.R		
	839.2400	1676.4654	2	73			K.NQLTSNPENTVFDAK.R		
	839.6600	1677.3054	2	(45)			K.NQLTSNPENTVFDAK.R		
	578.2100	1731.6082	3	32			K.KTKPYIQVDIGGGQTK.T		
	605.9000	1814.6782	3	(20)			R.IINEPTAAAIAYGLDKR.E		
	908.7200	1815.4254	2	30			R.IINEPTAAAIAYGLDKR.E		
	611.8300	1832.4682	3	34			K.NQLTSNPENTVFDAKR.L		
	612.1500	1833.4282	3	(24)			K.NQLTSNPENTVFDAKR.L		
	918.8000	1835.5854	2	58			K.SQIFSTASDNQPTVTIK.V		
	612.9000	1835.6782	3	(25)			K.SQIFSTASDNQPTVTIK.V		
	655.4700	1963.3882	3	33			K.KSQIFSTASDNQPTVTIK.V		
	1088.1800	2174.3454	2	(34)			K.LYGSAGPPPTGEEDTAEKDEL.-		
	1088.2500	2174.4854	2	65			K.LYGSAGPPPTGEEDTAEKDEL.-		
1053	443.6600	885.3054	2	20			R.RLSELLR.Y	10	238
	576.1600	1150.3054	2	44			K.YIDQEELNK.T		
	580.7000	1159.3854	2	33			K.SIYYITGESK.E		
	621.7300	1241.4454	2	47			K.ADLINNLGTIAK.S		
	625.1300	1248.2454	2	35			K.EQVANSAFVER.V		
	625.1300	1248.2454	2	(33)			K.EQVANSAFVER.V		
	683.2300	1364.4454	2	63			R.TLTLVDTGIGMTK.A + Oxidation (M)		
	757.2100	1512.4054	2	65			R.GVVDSEDLPLNISR.E		
697	403.6700	805.3254	2	16	12	11	R.NLGLYVK.T	11	204
	436.1600	870.3054	2	47			R.IDELEPR.R		
	437.1500	872.2854	2	40			R.LDLLEDR.S		
	442.1000	882.1854	2	21			R.AFFAGSQR.F		
	486.7300	971.4454	2	38			R.SLLLSVNAR.G		
	533.7200	1065.4254	2	41			K.NPEPELLVR.W		
	668.1900	1334.3654	2	14			R.SGGMERPFVLAR.A + Oxidation (M)		
	784.6100	1567.2054	2	47			K.DPAEGDGAQPEETPR.D		
	849.7300	1697.4454	2	54			R.DENSVELTMAEGPYK.I + Oxidation (M)		
	654.0900	1959.2482	3	30			K.MMDYLQGSGETPQTDVR.W + 2 Oxidation (M)		
	980.6700	1959.3254	2	(19)			K.MMDYLQGSGETPQTDVR.W + 2 Oxidation (M)		
	718.9700	2153.8882	3	11			R.VSQGSKDPAEGDGAQPEETPR.D		
624	540.1600	1078.3054	2	(38)	13	10	R.AFNDPFIQK.E	15	211
	540.1700	1078.3254	2	41			R.AFNDPFIQK.E		
	560.2000	1118.3854	2	55			R.FVVQNVSAQK.D		
	584.9300	1167.8454	2	1			K.VPSFFTDAER.R		
	661.2500	1320.4854	2	48			K.VLGTAFDPFLGGK.N		
	721.2000	1440.3854	2	50			K.DLLNMYIETEGK.M + Oxidation (M)		
	721.2000	1440.3854	2	(41)			K.DLLNMYIETEGK.M + Oxidation (M)		
	738.2400	1474.4654	2	73			K.DISTTLNADEAVAR.G		
	740.2200	1478.4254	2	54			R.AGGIETIANEFSDR.C		
	495.0800	1482.2182	3	20			K.QAYVDKLEELMK.I + Oxidation (M)		
	751.2600	1500.5054	2	91			K.VEDVSAVEIVGGATR.I		
	501.2100	1500.6082	3	(7)			K.VEDVSAVEIVGGATR.I		
	617.1200	1848.3382	3	11			R.LMNDMTAVALNYGIYK.Q + 2 Oxidation (M)		
388	415.7200	829.4254	2	20	16	14	K.LVELTQK.L	9	191
	421.7100	841.4054	2	42			R.LIITQVR.R		
	449.7000	897.3854	2	27			K.IIETPGIR.A		
	452.1900	902.3654	2	29			K.GETDLIQK.A		
	452.6900	903.3654	2	(16)			K.IPENIYR.G		
	452.6900	903.3654	2	22			K.IPENIYR.G		
	549.2200	1096.4254	2	48			K.IQEENVIPR.E		
	550.5600	1099.1054	2	34			K.EEAYNSLMK.S + Oxidation (M)		
	553.1400	1104.2654	2	(50)			R.LDSSAVLDTGK.Y		
	553.1700	1104.3254	2	54			R.LDSSAVLDTGK.Y		
	583.1600	1164.3054	2	64			K.GFTLNDAANSR.L		
	609.1600	1216.3054	2	46			K.MEEANIQPNR.V + Oxidation (M)		
	615.2300	1228.4454	2	58			K.GDVENIEVVQK.M		
	636.6900	1271.3654	2	54			K.TVLDQQQTPSR.L		
	692.2600	1382.5054	2	57			K.VIEEQLEPAVEK.I		
	848.2300	1694.4454	2	59			R.AGDMENAENILTVMR.D + 2 Oxidation (M)		
603	407.7300	813.4454	2	34	21	17	K.GSLVAPDR.L	18	182
	408.6800	815.3454	2	23			R.FIDFFK.R		
	450.7000	899.3854	2	(49)			R.LVSVLQNK.M		
	450.7200	899.4254	2	56			R.LVSVLQNK.M		
	473.7100	945.4054	2	46			R.SVLGEADQK.G		
	530.6700	1059.3254	2	57			R.GLEVTDDSPK.Y		
	543.7200	1085.4254	2	52			R.IVAVTGAEAQK.A		
	547.6500	1093.2854	2	48			K.SIDTGMGLER.L + Oxidation (M)		
	704.7600	1407.5054	2	42			R.AVFDETYPDPVR.V		
	717.6600	1433.3054	2	90			K.AEEIANEMIEAAK.A + Oxidation (M)		
	717.6800	1433.3454	2	(65)			K.AEEIANEMIEAAK.A + Oxidation (M)		
	738.7900	1475.5654	2	70			K.DIINEEEVQFLK.T		
	749.6700	1497.3254	2	70			K.VGAEDADGIDMAYR.V + Oxidation (M)		
	756.2100	1510.4054	2	65			K.GLVVDMDGFEEER.K + Oxidation (M)		
	521.4900	1561.4482	3	(29)			K.KAEEIANEMIEAAK.A + Oxidation (M)		
	521.5100	1561.5082	3	49			K.KAEEIANEMIEAAK.A + Oxidation (M)		
	533.8000	1598.3782	3	41			K.VDDSSEDKTEFTVK.N		
	533.8100	1598.4082	3	(22)			K.VDDSSEDKTEFTVK.N		
	571.8300	1712.4682	3	(57)			R.TITVALADGGRPDNTGR.G		
	571.8600	1712.5582	3	73			R.TITVALADGGRPDNTGR.G		
	835.6300	2503.8682	3	66			K.QFIDSNPNQPLVILEMESGASAK.A + Oxidation (M)		

401	414.7200	827.4254	2	31	29	21	R.VQEVLLK.G	27	227
	492.6700	983.3254	2	16			K.RPLKNDEL.-		
	524.1600	1046.3054	2	71			R.LAGLFNEQR.K		
	567.6600	1133.3054	2	35			R.TLGGLEMELR.L + Oxidation (M)		
	572.1000	1142.1854	2	54			K.DEPGEQVELK.E		
	630.6600	1259.3054	2	41			R.FFGDSAASMAIK.N + Oxidation (M)		
	630.6600	1259.3054	2	(37)			R.FFGDSAASMAIK.N + Oxidation (M)		
	643.6900	1285.3654	2	(19)			K.EAGMQPQLQIR.G + Oxidation (M)		
	643.7500	1285.4854	2	20			K.EAGMQPQLQIR.G + Oxidation (M)		
	432.5600	1294.6582	3	31			K.LPATEKPVLLSK.D		
	674.2200	1346.4254	2	52			R.SLAEDFAEQPIK.D		
	754.7200	1507.4254	2	37			K.AEAGPEGVAPAPEGEK.K		
	757.1400	1512.2654	2	29			R.AEPPLNASASDQGEK.V		
	761.2300	1520.4454	2	52			R.DAVVYPILVEFTR.E		
	546.1500	1635.4282	3	27			K.AEAGPEGVAPAPEGEKK.Q		
	561.8100	1682.4082	3	(38)			K.AANSLEAFIFETQDK.L		
	842.2500	1682.4854	2	(13)			K.AANSLEAFIFETQDK.L		
	842.2500	1682.4854	2	38			K.AANSLEAFIFETQDK.L		
	853.2700	1704.5254	2	23			K.DAVITVPVFFNQAER.R		
	868.2500	1734.4854	2	73			K.LGNTISSLFGGGTTPDAK.E		
	949.8000	1897.5854	2	65			K.LYQPEYQEVSTEEQR.E		
	1001.6800	2001.3454	2	30			K.EEAEAPVEDGSQPPPPEPK.G		
	668.1600	2001.4582	3	(8)			K.EEAEAPVEDGSQPPPPEPK.G		
	668.1800	2001.5182	3	(26)			K.EEAEAPVEDGSQPPPPEPK.G		
	1003.6700	2005.3254	2	(35)			K.VIPPAGQTEDAEPISEPEK.V		
	669.4700	2005.3882	3	39			K.VIPPAGQTEDAEPISEPEK.V		
	669.5300	2005.5682	3	(37)			K.VIPPAGQTEDAEPISEPEK.V		
	699.2600	2094.7582	3	47			K.VLQLINDNTATALSYGVFR.R		
	766.9100	2297.7082	3	42			R.LIPEMDQIFTEVEMTTLEK.V + 2 Oxidation (M)		
410	415.6600	829.3054	2	22	23	19	K.LVELTQK.L	14	238
	432.1100	862.2054	2	(15)			K.LDDLFLK.R		
	432.1900	862.3654	2	29			K.LDDLFLK.R		
	449.7200	897.4254	2	27			K.IIETPGIR.A		
	452.1500	902.2854	2	29			K.GETDLIQK.A		
	513.1600	1024.3054	2	23			K.FSPTDFLAK.M		
	514.2400	1026.4654	2	34			K.LQDAINILK.E		
	514.2900	1026.5654	2	(26)			K.LQDAINILK.E		
	528.6300	1055.2454	2	25			K.VYLQNEYK.F		
	549.1500	1096.2854	2	48			K.IQEENVIPR.E		
	553.1000	1104.1854	2	58			R.LDSSAVLDTGK.Y		
	556.1100	1110.2054	2	23			K.GAYDIFLNAK.E		
	583.0700	1164.1254	2	69			K.GFTLNDAANSR.L		
	583.1100	1164.2054	2	(63)			K.GFTLNDAANSR.L		
	609.1600	1216.3054	2	44			K.MEEANIQPNR.V + Oxidation (M)		
	615.2300	1228.4454	2	59			K.GDVENIEVVQK.M		
	636.7500	1271.4854	2	47			K.TVLDQQQTPSR.L		
	649.6300	1297.2454	2	73			K.DIQEESTFSSR.K		
	692.2200	1382.4254	2	55			K.VIEEQLEPAVEK.I		
	696.7900	1391.5654	2	58			K.MLNGLEDSIGLSK.M + Oxidation (M)		
	848.1100	1694.2054	2	62			R.AGDMENAENILTVMR.D + 2 Oxidation (M)		
	565.8000	1694.3782	3	(36)			R.AGDMENAENILTVMR.D + 2 Oxidation (M)		
	939.7700	1877.5254	2	52			K.TVQLTSSELESTLETLK.A		
414	414.6600	827.3054	2	(33)	48	25	R.VQEVLLK.A	35	204
	414.6700	827.3254	2	(22)			R.VQEVLLK.A		
	414.6900	827.3654	2	33			R.VQEVLLK.A		
	414.7300	827.4454	2	(16)			R.VQEVLLK.A		
	414.7500	827.4854	2	(22)			R.VQEVLLK.A		
	503.6600	1005.3054	2	26			R.EVQYLLNK.A		
	524.1200	1046.2254	2	70			R.LAGLFNEQR.K		
	524.1500	1046.2854	2	(61)			R.LAGLFNEQR.K		
	524.2400	1046.4654	2	(51)			R.LAGLFNEQR.K		
	567.6900	1133.3654	2	35			R.TLGGLEMELR.L + Oxidation (M)		
	567.7200	1133.4254	2	(35)			R.TLGGLEMELR.L + Oxidation (M)		
	572.1300	1142.2454	2	34			K.DEPGEQVELK.E		
	630.6800	1259.3454	2	44			R.FFGDSAASMAIK.N + Oxidation (M)		
	643.6300	1285.2454	2	(23)			K.EAGMQPQLQIR.G + Oxidation (M)		
	643.7100	1285.4054	2	(17)			K.EAGMQPQLQIR.G + Oxidation (M)		
	643.7300	1285.4454	2	(23)			K.EAGMQPQLQIR.G + Oxidation (M)		
	643.7300	1285.4454	2	27			K.EAGMQPQLQIR.G + Oxidation (M)		
	432.5300	1294.5682	2	34			K.LPATEKPVLLSK.D		
	674.2200	1346.4254	2	58			R.SLAEDFAEQPIK.D		
	754.7100	1507.4054	2	(37)			K.AEAGPEGVAPAPEGEK.K		
	503.4900	1507.4482	3	(32)			K.AEAGPEGVAPAPEGEK.K		
	503.4900	1507.4482	3	(29)			K.AEAGPEGVAPAPEGEK.K		
	754.7700	1507.5254	2	41			K.AEAGPEGVAPAPEGEK.K		
	757.1800	1512.3454	2	40			R.AEPPLNASASDQGEK.V		
	761.3000	1520.5854	2	(52)			R.DAVVYPILVEFTR.E		
	507.9200	1520.7382	3	54			R.DAVVYPILVEFTR.E		
	546.1800	1635.5182	3	25			K.AEAGPEGVAPAPEGEKK.Q		
	842.2800	1682.5454	2	60			K.AANSLEAFIFETQDK.L		
	574.9300	1721.7682	3	58			K.VAIVKPGVPMEIVLNK.E + Oxidation (M)		
	868.2900	1734.5654	2	65			K.LGNTISSLFGGGTTPDAK.E		
	668.1400	2001.3982	2	(21)			K.EEAEAPVEDGSQPPPPEPK.G		
	1001.7500	2001.4854	2	(40)			K.EEAEAPVEDGSQPPPPEPK.G		
	1001.7700	2001.5254	2	(37)			K.EEAEAPVEDGSQPPPPEPK.G		
	668.1900	2001.5482	3	41			K.EEAEAPVEDGSQPPPPEPK.G		
	1003.7700	2005.5254	2	47			K.VIPPAGQTEDAEPISEPEK.V		
	1003.7700	2005.5254	2	(43)			K.VIPPAGQTEDAEPISEPEK.V		
	669.5400	2005.5982	3	(45)			K.VIPPAGQTEDAEPISEPEK.V		
	669.5400	2005.5982	3	(32)			K.VIPPAGQTEDAEPISEPEK.V		
	698.8500	2093.5282	3	63			K.NINADEAAAMGAVYQAAALSK.A + Oxidation (M)		
	1047.8100	2093.6054	2	80			K.VLQLINDNTATALSYGVFR.R		
	1149.8300	2297.6454	2	55			R.LIPEMDQIFTEVEMTTLEK.V + 2 Oxidation (M)		
	766.9200	2297.7382	3	(38)			R.LIPEMDQIFTEVEMTTLEK.V + 2 Oxidation (M)		
	767.2500	2298.7282	3	(34)			R.LIPEMDQIFTEVEMTTLEK.V + 2 Oxidation (M)		
	781.1800	2340.5182	3	(21)			R.VESVFETLVEDSAEEESTLTK.L		
	781.1800	2340.5182	3	23			R.VESVFETLVEDSAEEESTLTK.L		
	846.2700	2535.7882	3	9			K.TVLSANADHMAQIEGLMDDVDFK.A + Oxidation (M)		
	865.1900	2592.5482	3	24			K.VETAGSEPGDTEPLELGGPGAEPEQK.E		
	1042.9300	3125.7682	3	32			K.DEPGEQVELKEEAEAPVEDGSQPPPPEPK.G		
608	407.6200	813.2254	2	18	14	11	K.GSLVAPDR.L	12	201
	450.6800	899.3454	2	(28)			R.LVSVLQNK.M		
	450.7200	899.4254	2	39			R.LVSVLQNK.M		
	473.6700	945.3254	2	46			R.SVLGEADQK.G		
	530.6600	1059.3054	2	56			R.GLEVTDDSPK.Y		
	543.7300	1085.4454	2	59			R.IVAVTGAEAQK.A		
	547.6300	1093.2454	2	54			K.SIDTGMGLER.L + Oxidation (M)		
	704.6700	1407.3254	2	(14)			R.AVFDETYPDPVR.V		
	704.6700	1407.3254	2	64			R.AVFDETYPDPVR.V		
	749.5900	1497.1654	2	63			K.VGAEDADGIDMAYR.V + Oxidation (M)		
	756.1900	1510.3654	2	66			K.GLVVDMDGFEEER.K + Oxidation (M)		

	521.5500	1561.6282	3	27			K.KAEEIANEMIEAAK.A + Oxidation (M)		
	521.5500	1561.6282	3	(17)			K.KAEEIANEMIEAAK.A + Oxidation (M)		
	533.8400	1598.4982	3	17			K.VDDSSEDKTEFTVK.N		
413	414.6500	827.2854	2	30	22	19	R.VQEVLLK.A	25	220
	414.7300	827.4454	2	(25)			R.VQEVLLK.A		
	524.2000	1046.3854	2	58			R.LAGLFNEQR.K		
	567.7200	1133.4254	2	35			R.TLGGLEMELR.L + Oxidation (M)		
	572.1400	1142.2654	2	30			K.DEPGEQVELK.E		
	643.6400	1285.2654	2	17			K.EAGMQPQLQIR.G + Oxidation (M)		
	432.6100	1294.8082	3	48			K.LPATEKPVLLSK.D		
	674.1800	1346.3454	2	50			R.SLAEDFAEQPIK.D		
	754.6900	1507.3654	2	45			K.AEAGPEGVAPAPEGEK.K		
	505.8700	1514.5882	3	28			K.TKEAGMQPQLQIR.G + Oxidation (M)		
	761.3000	1520.5854	2	30			R.DAVVYPILVEFTR.E		
	546.1700	1635.4882	3	59			K.AEAGPEGVAPAPEGEKK.Q		
	842.2700	1682.5254	2	41			K.AANSLEAFIFETQDK.L		
	574.8900	1721.6482	3	59			K.VAIVKPGVPMEIVLNK.E + Oxidation (M)		
	868.2300	1734.4454	2	64			K.LGNTISSLFGGGTTPDAK.E		
	868.6300	1735.2454	2	(5)			K.LGNTISSLFGGGTTPDAK.E		
	668.1100	2001.3082	3	14			K.EEAEAPVEDGSQPPPPEPK.G		
	1003.7600	2005.5054	2	(33)			K.VIPPAGQTEDAEPISEPEK.V		
	669.8400	2006.4982	3	41			K.VIPPAGQTEDAEPISEPEK.V		
	766.9000	2297.6782	3	35			R.LIPEMDQIFTEVEMTTLEK.V + 2 Oxidation (M)		
	789.7900	2366.3482	3	10			R.MVEEIGVELVVLDLPDLPEDK.L + Oxidation (M)		
	841.5500	2521.6282	3	45			K.VETGSEPGDTEPLELGGPGAEPEQK.E		

Supplemental Table 2S. RTqPCR primers	
**Gene**	**Oligonucleotides sequence**
SEC24A	5′-CTACACCAACGGTCCTGTCC-3′
	5′-TGGATTAGAGGCCACAGGTG-3′
SAR1B	5′-TGGATTAGAGGCCACAGGTG-3′
	5′-CATGCCAGCAATGGTCAGTT-3′
GAPDH	5′-ACCACAGTCCATGCCATCAC-3′
	5′-TCCACCACCCTGTTGCTGTA-3′
HYOU1	5′-GCAGACCTGTTGGCACTGAG-3′
	5′-TCACGATCACCGGTGTTTTC-3′
HSPH1	5′-CAGTCTCACCATTTGCATCAC-3′
	5′-CCTCTTGAACAAGCAGTACAG-3′
HSP90AB1	5′-AGAAATTGCCCAACTCATGTCC-3′
	5′-ATCAACTCCCGAAGGAAAATCTC-3′
HSPB1	5′-TGGACCCCACCCAAGTTTC-3′
	5′-CGGCAGTCTCATCGGATTTT-3′
ATP5B	5′-AAACAATTTGCTCCCATTCATGC-3′
	5′-GACAACCTTGATACCAGTCACC-3′
LRPPRC	5′-GCTCATAGGATATGGGACACACT-3′
	5′-CCAGGAAATCAGTTGGTGAGAAT-3′
MRI1	5′-CAGGAGCTGACCGATGTTAATG-3′
	5′-CCACCAGTGATGAGGTCGT-3′
EEF1D	5′-CCACCAGTGATGAGGTCGT-3′
	5′-ATCCCAAGGCTTGACATCCAG-3′
EIF4H	5′-GCATAAGGAGTGTACGGCTAGT-3′
	5′-CCATCGTATGTCAAGGCTTCC-3′
EEF1G	5′-TGTTTGAGAGCAACGCCATTG-3′
	5′-ATCGGAATCAGCAAAGCTCAC-3′
ERP29	5′-CCTGGATACGGTCACTTTCTACA-3′
	5′-AGTTTTCAGCAAGACGCTTGA-3′
COPE	5′-CAGAGGAAGTTCGGTGTGGTC-3′
	5′-GGGCGAGGTAGTCAGCAAAC-3′
RPLP0	5′-AGCCCAGAACACTGGTCTC-3′
	5′-ACTCAGGATTTCAATGGTGCC-3′
HSPD1	5′-CTACTGTACTGGCACGCTCTA-3′
	5′-CAACAGCTAACATCACACCTCTC-3′
HSPA1A/B	5′-GCTTCAAGACTTTGCATTTCC-3′
	5′-AAAGCAAGTTCAGTACTTCACC-3′

Chip-DNA primers	
	**Oligonucleotides sequence**
**SAR1B-SEC24A regulatory element**	5′-TGCTTAAATAGCACAAAGTTC-3′
	5′-GAAGAGGAAATAGAGCTCAGA-3′
**GAPDH exon**	5′-CGGAGTCAACGGATTTGGTCGTAT-3′
	5′-AGCCTTCTCCATGGTGGTGAAGAC-3′
